# Supplementary material for: Tracing the Evolution of the p53 Tetramerization Domain
Source: Structure. 2014 Sep 2;22(9):1301–10. doi: 10.1016/j.str.2014.07.010 (PMC4155161; doi:10.1016/j.str.2014.07.010)
Supplement: Document S1. Figures S1 and S2 and Tables S1–S3 [file mmc1.pdf]

Structure, Volume *22*

Supplemental Information

Tracing the Evolution of the p53 Tetramerization Domain

Andreas C. Joerger, Rainer Wilcken, and Antonina Andreeva

Table S1, related to Figures 2 and 6.

Consensus secondary structure prediction of p53 C-terminal regions in vertebrates

Species	Consensus secondary structure prediction*
Human<br>*Homo sapiens*	GEYFTLQIRGRERFEMFRELNEALELKDAQ**A**GKEPGGSRAHSSHLKSKKGQSTSRHKKLMFKTEGPDSD<br>cceeeeeecchhhhhhhhhhhhhhhhhhhhccccccccccc?ccccccccccccchheeeeeccccccc
Dolphin<br>*Delphinapterus leucas*	GEYFTLQIRGRERFEMFRELNEALELKDAQ**A**GKEPGESRAHSSHLKSKKGQSPSRHKKLMFKREGPDSD<br>cceeeeeecchhhhhhhhhhhhhhhhhhhhhcccccccccccccccccccccccchhh??eeccccccc
Mouse<br>*Mus musculus*	GEYFTLKIRGRKRFEMFRELNEALELKDAH**A**TEESGDSRAHSSYLKTKKGQSTSRHKKTMVKKVGPDSD<br>cceeeeeeccchhhhhhhhhhhhhhhhhhhhhhcccccc?ch?hhcccccccccchhheeeeccccccc
Chicken<br>*Gallus gallus*	NEIFYLQVRGRRRYEMLKEINEALQLAEGG**S**APRPSKGRRVKVEGPQPSCGKKLLQKGSD<br>cceeeeeecc?hhhhhhhhhhhhhhhhcccccccccccceeee?cccccccc?hhccccc
African clawed frog<br>*Xenopus laevis*	EEIFTLRIKGRSRYEMIKKLNDALELQESL**D**QQKVTIKCRKCRDEIKPKKGKKLLVKDEQPDSE<br>c?eeeeeecccchhhhhhhhhhhhhhhhhhhhhh???e?h?ccccccccccceeeeeccccccc
Axolotl<br>*Ambystoma mexicanum*	EETFTLQIHGRERYEMFKKLNEALELKDMI**P**QTDLEKIKQQKSQKTKKERDPPAPKNGKKLLVKDETQ<br>cceeeeeeccchhhhhhhhhhhhhhhhhcccc?hhhhhhhhhh???ccccccccccccceeeeecccc
Coelacanth<br>*Latimeria chalumnae*	EELYSLQVRGRERYEMFKKLNNALELQDSV**S**AAETEKHKTKPSKNASRKELEPKKGKKLLVKEEGSGGG<br>c?h?eeeecc?hhhhhhhhh?hhhhhhhhhhhhhhhhcccccccch?hhhccccccceeeeeecccccc
Elephant shark<br>*Callorhinchus milli*	TEVFTLQVTGRERYETLKQINESLEVQELV**P**ASVVQACRQQHKLRLKAAHKKESSASEPKKGRKLPLKDEVDSE<br>ceeeeeeecc?hhhhhhhhhhhhhhhhhhcc?hhhhhhhhhhhhhhhhhhcccccccccccccccccccccccc
Spotted gar<br>*Lepisosteus oculatus*	EEIFTLQVRGRERFEMLKKINESLELKDLV**P**VADLEKYRQKLHTRSSSRREKDKEKEPKKGKKLLVKEEKTDSD<br>cceeeeeecc?hhhhhhhhhhhhhhh?ccccchhhhhhhhhhccccchhhh?cccccccccceeeeeecccccc
Zebrafish<br>*Danio rerio*	EEIFTLQVRGRERYEILKKLNDSLELSDVV**P**ASDAEKYRQKFMTKNKKENRESSEPKQGKKLMVKDEGRSDSD<br>c?eeeeeecc?hhhhhhhhhccccccccccccchhhhhhhhhhh?ccccccccccccccceeeeecccccccc
Barbel<br>*Barbus barbel*	EEIYTLQVRGKERYEMLKKINDSLELSDVV**P**PSEMDRYRQKLLTKGKKKDGQTPEPKRGKKLMVKDEKSDSD<br>cceeeeeecc?hhhhhhhhhhcc?h?ccccccc?hhhhhhhhhhcccccccccccccccceeeeeccccccc
Channel catfish<br>*Ictalurus punctatus*	DEIYTLQVRGKERYEFLKKINDGLELSDVV**P**PADQEKYRQKLLSKTCRKERDGAAGEPKRGKKRLVKEEKCDSD<br>cceeeeeecc?hhhhhhhhhcccccccccccccchhhhhhhhhhhhhh?hcccccccccccc?eeeeccccccc
Walking catfish<br>*Clarias batrachus*	EEIFLLQIRGRDRFNMLKTINDSLELMDMM**P**AADKDKYRQKRTLKNLKKRPGCGKKTAPKR<br>cceeeeeecccchhhhhhhhhhhhhhhhhcchhchhhhhhhhhhhhhcccccccccccccc
Atlantic salmon<br>*Salmo salar*	DEIYTLQIRGKEKYEMLKKFNDSLELSELV**P**VADADKYRQKRLTKRVAKREIGVGPKKGKKLLVKEEKSDSD<br>cceeeeeeccchhhhhhhhhcchhhhhh?ccchhhhhhhhhhhhhhhhhhhccccccccceeeeeecccccc
Rainbow trout<br>*Oncorhynchus mykiss*	DEIYTLQIRGKEKYEMLKKFNDSLELSELV**P**VADADKYRQKCLTKRVAKRDFGVGPKKRKKLLVKEEKSDSD<br>cceeeeeeccc?hhhhhhhhcchhhhhh?ccccc?hhhhhhhhhhhhh?ccccccccc?cheeeeccccccc
European whitefish<br>*Coregonus lavaretus*	DEIYTLQIRGKEKYEMLKKLNDCLELSELV**P**AADADKYRQKRLTKRVAKRELGVGPKKGKKLLVKEERSDSD<br>cceeeeeeccchhhhhhhhhhhhhhhhhhccc?hhhhhhhhhhhhhhhhhhccccccccceeeeeccccccc
Northern pike<br>*Esox lucius*	DEIYTLRIRGKERYEMLKKINDGLELSDLV**P**TADADKYRQKQDPLTKRVAKQDFRVGPKKGKKLLVKEERSDSD<br>cceeeeeecc?hhhhhhhhhccccc?cccccccchhhhhhccc?hhhhhhhhcccccccccceeeeeccccccc
Rainbow smelt<br>*Osmerus mordax*	EEVFTLQVRGKERFEILKKINDGLELSDLV**P**AADAERYRQKPPAKSTKKEKDGPGVPKKGKRLLVKEEKSD<br>cceeeeeccccchhhhhhhhccccchchhcchhhhhhhhcccccccccccccccccccccceeeeeccccc
Atlantic cod<br>*Gadus morhua*	KEVFVLQVVGRKRYEVLKQINDALALQERM**K**VKQEVQGGPSRGKRRLGDRTDEGTD<br>cceeeeee?cchhhhhhhhhhhhhhhhhhhhhhhhhcccccccccccccccccccc
Japanese rice fish<br>*Oryzias latipes*	REVFHFEVYGRERYEFLKKINDGLELLEKE**S**KSKNKDSGMVPSSGKKLKSN<br>cheeeeeeccchhhhhhhhhcchhhhhhhhccccccccccccccccccccc
Turquoise killifish<br>*Nothobranchius furzeri*	KEMIPLYIQGRKKWNLMKRISDGLDLVEKE**K**KQLLVQEVLPTSGKRLLKKDRSDSD<br>ccccceeecccchhhhhhhhhc?hhhhhhhhhhhhhhh?ccccchhhhhccccccc
Green swordtail<br>*Xiphophorus helleri*	KEIYTLSIRGRNRYLWFKSLNDGLELMDKT**G**PKIKQEIPAPSSGKRLLKGGSDSD<br>ceeeeeeecccc?eeeehhhcc?hhhhhccccccc?ccccccccc??eccccccc
Pufferfish<br>*Takifugu rubripes*	KELFTLQIRGRKRYEMLKKINDGLELLENK**P**KCKAAAKPECPVPPRGKRLLHRGEKSDSD<br>cc??ee??cchhhhhhhhhhcc?hhhhhccccccccccccccccccccceeecccccccc
Three-spined stickleback<br>*Gasterosteus aculeatus*	KDVFVLRVRGRERYEWLKKIHDGLELLDRE**R**LSKPTVSVKHEAAAPSGGKRLLRSDSE<br>cceeeeeeccchhhhhhhhhhchhhhhhhhcccccc?eeeeccccccccceeeecccc
Orange-spotted grouper<br>*Epinephelus coioides*	KDVFVLHVRGRERFEMLKKINDGLELLDKE**S**KTKTKVSVKHELAVPSSGKRLPHRGERSDSD<br>cceeeee?cc?hhhhhhhhhcc?hhhh?hcccccceeeeeeeeecccccccccccccccccc
Mangrove killifish<br>*Kryptolebias marmoratus*	KEVFTLHVRGRERYEMLKKINAGLELLDTD**G**KRTKQEKKFPKKDKSDSD<br>ceeeeeeecc?hhhhhhhhhhccc??hcccccccc??cccccccccccc
Climbing perch<br>*Anabas testudineus*	KDVFVLHVRGRDRFEMLKKINDGLELLDKK**N**KTKSKASTKHGVPVPCSGKRLLQRGDRSDSD<br>cceeeeeeccc?hhhhhhhhcc?hhhhhccccccccccccccccccccccc??ccccccccc
Olive flounder<br>*Paralichthys olivaceus*	KEVFHLPIVGRGRYEMFKKINEGLELLDRE**K**TKKVPVKQELPVPSTGKRLLQRGEQSDSD<br>ceeeeeecccc?hhhhhhhhhhhhhhhhhcccccccccccccccccc?hhh?cccccccc

*Consensus secondary structure prediction (DPM, DSC, GOR IV. HNN, PHD, Predator, SIMPA96, SOPM) using Network Protein Sequence Analysis (Combet *et al*., 2000). h = α-helix, e = β-strand, c = coil, ? = ambiguous. The residue corresponding to the N-terminal cap of helix H2 in the tetramerization domain of human p63 and p73 is in bold and underlined. Names of ray-finned fishes are highlighted in different colors: green, Acanthomorpha; blue, other ray-finned fishes.

Table S2, related to Figures 2 and 6.

Accession codes of selected vertebrate p53 family proteins

Species	Genome sequenced	Protein	Database	Accession code	Expression
Human	yes	p53	UniProt	P04637	yes
Homo sapiens		p63	UniProt	Q17RN8	yes
		p73	UniProt	Q9H3D4	yes
Mouse	yes	p53	UniProt	Q549C9	yes
Mus musculus		p63	UniProt	O88898	yes
		p73	UniProt	Q9JJP2	yes
Rabbit	yes	p53	UniProt	Q95330	yes
Oryctolagus cuniculus		p63	Ensembl	G1TBP7	predicted
		p73	Ensembl	G1U940 fragment	predicted
Dog	yes	p53	UniProt	Q29537	yes
Canis familiaris		p63	Ensembl	ENSCAFT00000022170	predicted
		p73	Ensembl	ENSCAFT00000030985	predicted
Elephant	yes	p53	UniProt	G3T035	predicted
Loxodonta africana		p63	UniProt	G3TT62	predicted
		p73	UniProt	G3SZA7	predicted
Chicken	yes	p53	UniProt	P10360	yes
Gallus gallus		p63	UniProt	Q9DEC7	yes
		p73	Ensembl	ENSGALT00000001487	predicted[1]
Zebra finch	yes	p53	GenBank	EE060750 (EST)	yes[2]
Taeniopygia guttata		p63	Ensembl	ENSTGUT00000009791	predicted
		p73	Ensembl	ENSTGUT00000002858	predicted
African clawed frog	yes	p53	UniProt	Q6NTF1	yes
Xenopus tropicalis		p63	Ensembl	ENSXETT00000055541	predicted
		p73	Ensembl	ENSXETT00000010981	predicted
Axolotl	no	p53	Uniprot	Q0GMA7	yes
Ambystoma mexicanum		p63	-	-	-
		p73	-	-	-
Coelacanth	yes	p53	UniProt	H3B1Z4	predicted
Latimeria chalumnae		p63	UniProt	H3B2L6	predicted
		p73	UniProt	H3BII1	predicted
Elephant shark	yes	p53	UniProt	G9J1L8	yes
Callorhinchus milli		p63	UniProt	G9J1L9	yes
		p73	UniProt	G9J1M0	yes
Spotted gar	yes	p53	Ensembl	ENSLOCG00000013832[3]	predicted
Lepisosteus oculatus		p63	Ensembl	ENSLOCG00000005148[3]	predicted
		p73	Ensembl	ENSLOCT00000006351	predicted
Zebrafish	yes	p53	UniProt	G1K2L5/Q502Q9	yes
Danio rerio		p63	UniProt	A7YYJ7	yes
		p73	UniProt	Q801Z7	yes
Barbel	no	p53	UniProt	Q9W678	yes
Barbus barbus		p63	-	-	-
		p73	UniProt	Q9W664	yes
Channel catfish	no	p53	Uniprot	O93379	yes
Ictalurus punctatus		p63	-	-	-
		p73	-	-	-
Yellow catfish	no	p53	UniProt	F5A7P3	yes
Tachysurus fulvidraco		p63	-	-	-
		p73	-	-	-
Walking catfish	no	p53	GenBank	GW787457 (EST)	yes
Clarias batrachus		p63	-	-	-
		p73	-	-	-
Atlantic salmon	no	p53	Uniprot	C0H8X1	yes
Salmo salar		p63	-	-	-
		p73	-	-	-

Table S2 (continued)

Accession codes of selected vertebrate p53 family proteins

Species	Genome sequenced	Protein	Database	Accession code	Expression
Rainbow trout *Oncorhynchus mykiss*	no	p53	Uniprot	P25035	yes
		p63	-	-	-
		p73	-	-	-
European whitefish *Coregonus lavaretus*	no	p53	Uniprot	B5TJK8	yes
		p63	-	-	-
		p73	-	-	-
Northern pike *Esox lucius*	no	p53	GenBank	GH247610 (EST)	yes
		p63	-	-	-
		p73	-	-	-
Rainbow smelt *Osmerus mordax*	no	p53	GenBank	EL541113 (EST)	yes
		-	-	-	-
		-	-	-	-
Atlantic cod *Gadus morhua*	yes	p53	GenBank	GW859200 (EST)[4]	yes
		p63	Ensembl	ENSGMOT00000000426	predicted[5]
		p73	Ensembl	ENSGMOT00000017093	predicted
Medaka *Oryzias latipes*	yes	p53	UniProt	P79820	yes
		p63	UniProt	H2MLN6	predicted
		p73	UniProt	H2LHQ7	predicted
Turquoise killifish *Nothobranchius furzeri*	no	p53	UniProt	B3TLB0	yes
		p63	-	-	-
		p73	-	-	-
Tilapia *Oreochromis niloticus*	yes	p53	UniProt	D5KTJ0 (I3KRX9 error)	yes
		p63	UniProt	I3KT80	predicted
		p73	UniProt	I3J187	predicted
Southern platyfish *Xiphophorus maculatus*	yes	p53	UniProt	Q92143	yes
		p63	Ensembl	ENSXMAP00000016461	predicted
		p73	Ensembl	ENSXMAP00000017403	predicted
Three-spined stickleback *Gasterosteus aculeatus*	yes	p53	UniProt	G3Q6V4	predicted
		p63	UniProt	G3PK82	predicted[4]
		p73	UniProt	G3NTK8	predicted
Mangrove killifish *Kryptolebias marmoratus*	no	p53	UniProt	A9XR54	predicted
		p63	-	-	-
		p73	-	-	-
Olive flounder *Paralichthys olivaceus*	no	p53	UniProt	A5JSV4	yes
		p63	-	-	-
		p73	-	-	-
Orange-spotted grouper *Epinephelus coioides*	no	p53	UniProt	F8RKR1	yes
		p63	-	-	-
		p73	-	-	-
Climbing perch *Anabas testudineus*	no	p53	UniProt	R9XXS5	yes
		p63	-	-	-
		p73	-	-	-
Pufferfish *Takifugu rubripes*	yes	p53	UniProt	H2U134	predicted
		p63	UniProt	H2S6K3	predicted
		p73	UniProt	H2UMJ4	predicted
Green spotted puffer *Tetraodon nigroviridis*	yes	p53	UniProt	H3CXQ0	predicted
		p63	UniProt	H3D8D5	predicted
		p73	UniProt	H3D350	predicted

[1] Prediction of a transcript containing all 14 exons but with a stop codon after residue 213.
[2] Not predicted from current genome assembly
[3] Gene has two transcripts
[4] Missing C-terminal half in the p53 protein predicted from the current version of the genome assembly (ENSEMBL release 74, December 2013)
[5] SAM domain missing

Table S3, related to Figure 2.

Accession codes of p53 family proteins in selected non-vertebrate genomes

Species	Database	Accession code	Expression	Length	SAM domain
Sea squirt *Ciona intestinalis*	UniProt	Q4H300	yes	489	no
	UniProt	Q4H301	yes	415	no
	UniProt	Q4H2Z8[1]	yes	419	no
Lancelet *Branchiostoma floridae*	UniProt	C3YXH3	predicted	416	no
	UniProt	C3XPU2	predicted	649	yes
Soft-shell clam *Mya arenaria*	UniProt	Q9NGC8/Q9NGC7[2]	yes	621/443	yes/no
Blue mussel *Mytilus edulis*	UniProt	Q1AMZ8	yes	657	yes
Pacific oyster *Crassotrea gigas*	UniProt	K1RC48	predicted	790	yes
Sea snail *Lottia gigantea*	Ensembl	LotgiT182533	predicted	607	yes
Bobtail squid *Euprymna scolopes*	UniProt	Q0H3B6	yes	566	yes
Polychaete worm *Capitella teleta*	Ensembl	CapteT137251	predicted	573	yes
Leech *Helbodella robusta*	Ensembl	HelroT167604	predicted	383	no
Sea urchin *Strongylocentrotus purpuratus*	UniProt	H9N2D2/H9N2D3[2]	yes	691/458	yes/no
Roundworm *Caenorhabditis elegans*	UniProt	Q20646	yes	644	yes
Fruit fly *Drosophila melanogaster*	UniProt	Q9N6D8	yes	385	no
Blood fluke *Schistosoma mansoni*	UniProt	G4LYM1	predicted	696	no
	UniProt	G4VAQ1	predicted	410	no
Starlet sea anemone *Nematostella vectensis*	UniProt	A8DPD6	yes	492	no
	UniProt	A7SFL1	predicted	497	no
	UniProt	A7S162	predicted	203	no
Placozoa *Trichoplax adhaerens*	UniProt	B3RZS6	predicted	576	yes
Sponge *Amphimedon queenslandica*	UniProt	I1FGP9	predicted	484	yes
	UniProt	I1FPX8	predicted	332	no
Amoeba *Capsaspora owczarzaki*	UniProt	E9BW10	predicted	730	yes
Choanoflagellate *Monosiga brevicollis*	UniProt	A9V4M3	predicted	571	no
	UniProt	A9UZX3	predicted	523	yes

[1]Gene has no introns
[2]Two different splice variants of a single p63/p73-like gene

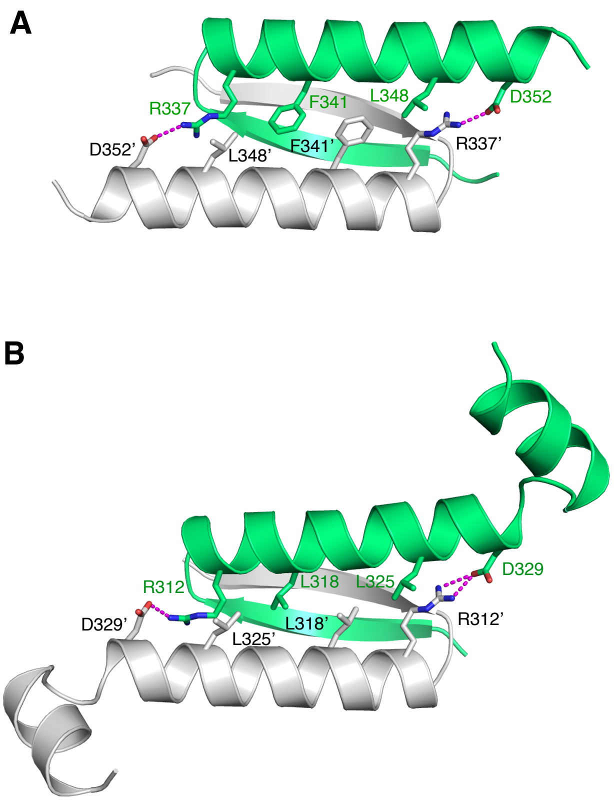

Figure S1, related to Figures 1 and 5. Conserved intermolecular salt bridge in p53 tetramerization domains. (A) Primary dimer in the structure of human p53 tetramerization domain (PDB entry 1C26). The two chains are shown as ribbon diagrams, and selected side chains at the dimer subunit interface are shown as stick models. The second dimer of the tetramer is omitted for clarity. Dimers are stabilized via an intermolecular β-sheet, hydrophobic interactions and a highly conserved salt bridge between Arg337 and Glu352. (B) Primary dimer in the zebrafish p53 tetramerization domain showing conservation of the stabilizing intermolecular salt bridge. The view is the same as in panel A. The central phenylalanine in the human tetramerization domain is replaced by a leucine in the zebrafish protein. Interestingly, there is a slight but systematic deviation from perfect 2-fold symmetry in the packing of the H1 helices within a primary dimer of the zebrafish protein. In all three independent primary dimers of crystal form I, the distance between the Cα atoms of Arg314 and Leu325 from adjacent helices is 7.2 Å at one end of the dimer and 8.7 Å at the other end. Essentially the same difference is also observed in the six independent dimers in the asymmetric unit of crystal form II.

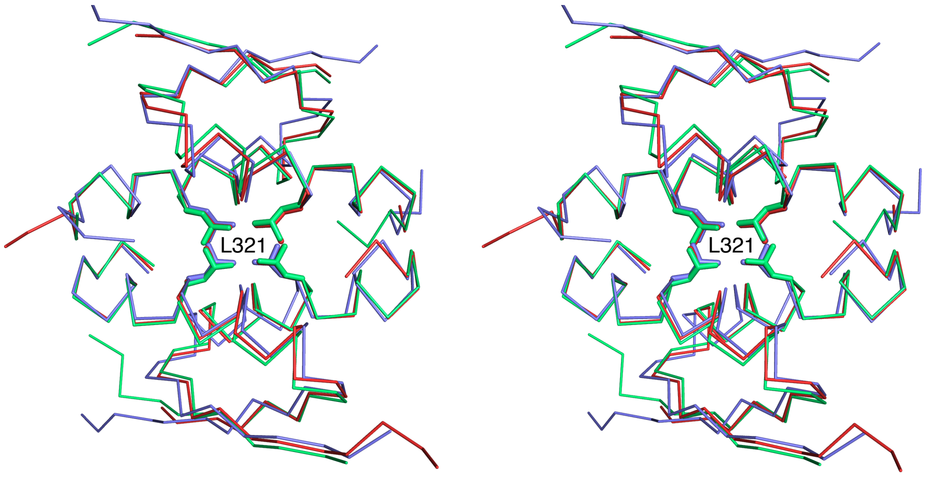

Figure S2, related to Figure 5. Crystal structures of a truncated zebrafish p53 tetramerization domain lacking helix H2. Tetramers observed in three different crystal forms were superimposed and are shown as Cα-traces, with the Leu321 side chains at the center of the tetramer interface shown as stick models. The tetramer in the orthorhombic crystal form I is shown in red, the tetramer in the trigonal crystal form II in green and one of the two tetramers in the monoclinic crystal form III (chains A to D) in blue (see Table 1).
