# Supplementary material for: Tracing the Evolution of the p53 Tetramerization Domain
Source: Structure. 2014 Sep 2;22(9):1301–10. doi: 10.1016/j.str.2014.07.010 (PMC4155161; doi:10.1016/j.str.2014.07.010)
Supplement: Document S2. Article plus Supplemental Information [file mmc2.pdf]

Structure
Article

Tracing the Evolution of the p53 Tetramerization Domain

Andreas C. Joerger,[1,*] Rainer Wilcken,[1] and Antonina Andreeva[1]
[1]MRC Laboratory of Molecular Biology, Francis Crick Avenue, Cambridge CB2 0QH, UK
*Correspondence: acj2@mrc-lmb.cam.ac.uk
http://dx.doi.org/10.1016/j.str.2014.07.010
This is an open access article under the CC BY license (http://creativecommons.org/licenses/by/3.0/).

SUMMARY

The tetrameric transcription factors p53, p63, and p73 evolved from a common ancestor and play key roles in tumor suppression and development. Surprisingly, p63 and p73 require a second helix in their tetramerization domain for the formation of stable tetramers that is absent in human p53, raising questions about the evolutionary processes leading to diversification. Here we determined the crystal structure of the zebrafish p53 tetramerization domain, which contains a second helix, reminiscent of p63 and p73, combined with p53-like features. Through comprehensive phylogenetic analyses, we systematically traced the evolution of vertebrate p53 family oligomerization domains back to the beginning of multicellular life. We provide evidence that their last common ancestor also had an extended p63/p73-like domain and pinpoint evolutionary events that shaped this domain during vertebrate radiation. Domain compaction and transformation of a structured into a flexible, intrinsically disordered region may have contributed to the expansion of the human p53 interactome.

INTRODUCTION

The p53 family of transcription factors comprises three family members in vertebrates (p53, p63, and p73), which originated from a common ancestral gene (Belyi et al., 2010). The three paralogs execute overlapping and distinct functions in the cell cycle. p53 is a key tumor suppressor (Lane, 1992; Levine et al., 2011; Vousden and Prives, 2009), and p63 and p73 play major roles in development, differentiation, and germ-line protection (Dötsch et al., 2010). The evolutionary history of the p53 family goes back more than 1 billion years to the beginning of multicellular life. p53 family proteins have, for example, been found in modern-day descendants of the early metazoan sea anemone, where their role is to protect the germ-line from DNA damage (Belyi et al., 2010; Pankow and Bamberger, 2007). p53 family genes are also found in the genome of choanoflagellates, the closest unicellular relatives to animals (King et al., 2008; Nedelcu and Tan, 2007), and placozoans (Lane et al., 2010). Vertebrates generally have all three family members, whereas *Drosophila melanogaster*, the nematode *Caenorhabditis elegans*, and many molluscs only have a single homolog. There are, however, a number of invertebrate lineages, such as sea anemones and mosquitoes, where independent gene duplications have occurred (Belyi et al., 2010; Nedelcu and Tan, 2007). A recent analysis of the genome of the elephant shark (*Callorhinchus milii*) has confirmed the presence of all three paralogs, suggesting that the two gene duplications, giving rise to the three family members present in modern-day vertebrates, occurred early in vertebrate evolution, before the emergence of cartilaginous fishes in the Silurian period (Lane et al., 2011).

The p53 family proteins have a modular domain organization comprising DNA-binding and tetramerization domains that are linked and flanked by intrinsically disordered regions with high sequence diversity (Dötsch et al., 2010; Joerger and Fersht, 2008; Xue et al., 2013) (Figure 1). p63 and p73 have an extended C-terminal region with a sterile alpha motif (SAM) domain, a putative protein interaction module that is absent in p53 (Dötsch et al., 2010). All three family members form tetramers in their active form (Brandt et al., 2009; Luh et al., 2013). A notable exception is the p53 homolog from *Caenorhabditis elegans*, which forms dimers and has a unique oligomerization domain that includes a SAM domain (Ou et al., 2007). Human p53 tetramerizes via a short hairpin motif in its C-terminal region that consists of a β strand followed by an α helix. The overall architecture of the tetramer is a dimer of primary dimers, stabilized via an intermolecular β sheet and predominantly hydrophobic packing of the α helices (Clore et al., 1995; Jeffrey et al., 1995; Lee et al., 1994). This structure was long thought to be the hallmark of all p53 family members in vertebrates, but more recent structural studies have shown that human p63 and p73 require an additional helix to form stable and transcriptionally active tetramers (Figures 1B and 1C) (Coutandin et al., 2009; Joerger et al., 2009; Natan and Joerger, 2012). The *Drosophila* p53 homolog also has an extended tetramerization domain with an additional helical segment, but the additional helix forms fundamentally different packing interactions than observed in the tetramers of human p63 and p73 with little overall sequence conservation (Ou et al., 2007). The structural differences between the human p53 family members raise questions about the nature of the oligomerization domain in the common ancestral protein and the mechanisms and functional implications of divergent evolution of the p53 protein.

Here, we have traced the evolutionary history of the p53 tetramerization domain through systematic genome analyses. Crystallographic studies on the zebrafish p53 tetramerization domain then confirmed the presence of the p63/p73 signature helix in p53 proteins of a subgroup of bony fishes, providing evidence that the last common ancestor of p53, p63, and p73 also

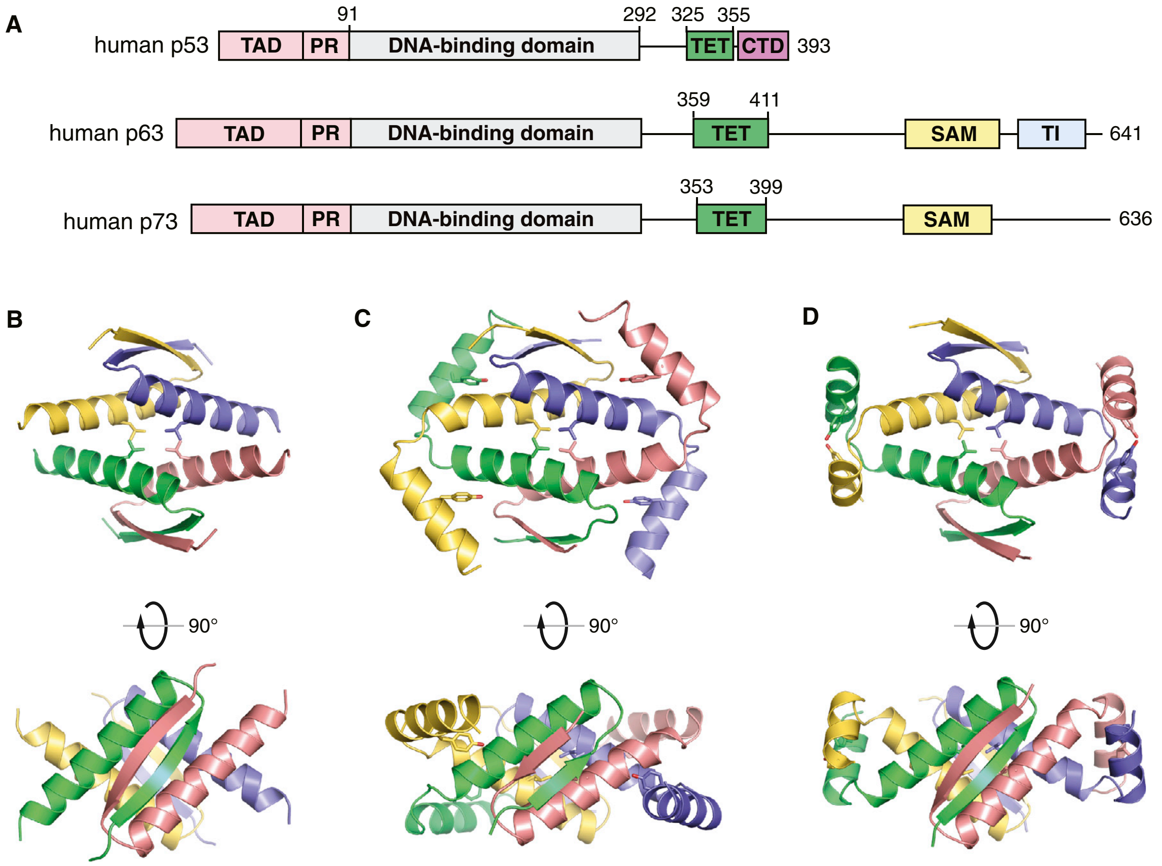

Figure 1. p53 Family Tetramerization Domains

(A) Domain organization of human p53 family members (Dötsch et al., 2010; Joerger and Fersht, 2008). The three proteins are aligned on the basis of the highly conserved DNA-binding domain. The location of the tetramerization domain (TET) is highlighted in green. The N-terminal transactivation domain (TAD) and proline-rich region (PR) are intrinsically disordered. The C-terminal regulatory domain (CTD) of p53 is also intrinsically disordered and interacts with a multitude of protein partners. In contrast, p63 and p73 have an extended C-terminal region containing a SAM domain. p63 has an additional transactivation inhibitory domain (TI) that plays a role in stabilizing a latent dimeric form (Deutsch et al., 2011).

(B) Crystal structure of the human p53 tetramerization domain (PDB entry 1C26).

(C) Crystal structure of the human p73 tetramerizaton domain (PDB entry 2WQI).

(D) Crystal structure of the zebrafish p53 tetramerization domain determined in this study. All three tetramerization domain structures are shown in two orientations, with individual subunits in different colors.

See also Figure S1.

had an extended domain with an additional helix that was subsequently lost in many modern-day p53 proteins as a result of divergent evolution. This process may have had important functional implications by contributing to the expansion of the human p53 interaction network and fine-tuning of its regulatory circuits.

RESULTS AND DISCUSSION

Sequence Analyses of Vertebrate p53 Family Oligomerization Domains

To trace the evolution of the p53 tetramerization domain from a potentially p63/p73-like ancestral protein with an extended domain, we analyzed the currently available sequences of vertebrate p53 family members and searched for sequences with a putative second helix as observed in p63 and p73 (Figure 2). The characteristic features of this helix in p63 and p73 are a strictly conserved proline at the N-terminal cap of the helix that is preceded by two hydrophobic residues (key for positioning the helix to engage in stabilizing interactions with a neighboring subunit), a hydrophobic side chain at position N+4, and a conserved YRQ motif at position N+7, followed by a varying number of glutamines. In the currently available p53 sequences of mammals and birds, this motif is absent. In fish and amphibians, however, we found species both with and without a putative second helix. The tetramerization domain of the elephant shark, a member of the cartilaginous fishes, retains key features of the second helix in p63/p73, but the usually conserved tyrosine that forms essential subunit contacts is replaced by a cysteine. Consensus secondary structure prediction suggested that this region forms an α helix (Table S1 available online). Among the group of ray-finned bony fishes, there are numerous species with the signature motif of a second, p63/p73-like helix at the end of the canonical p53 tetramerization domain region (Figure 2),

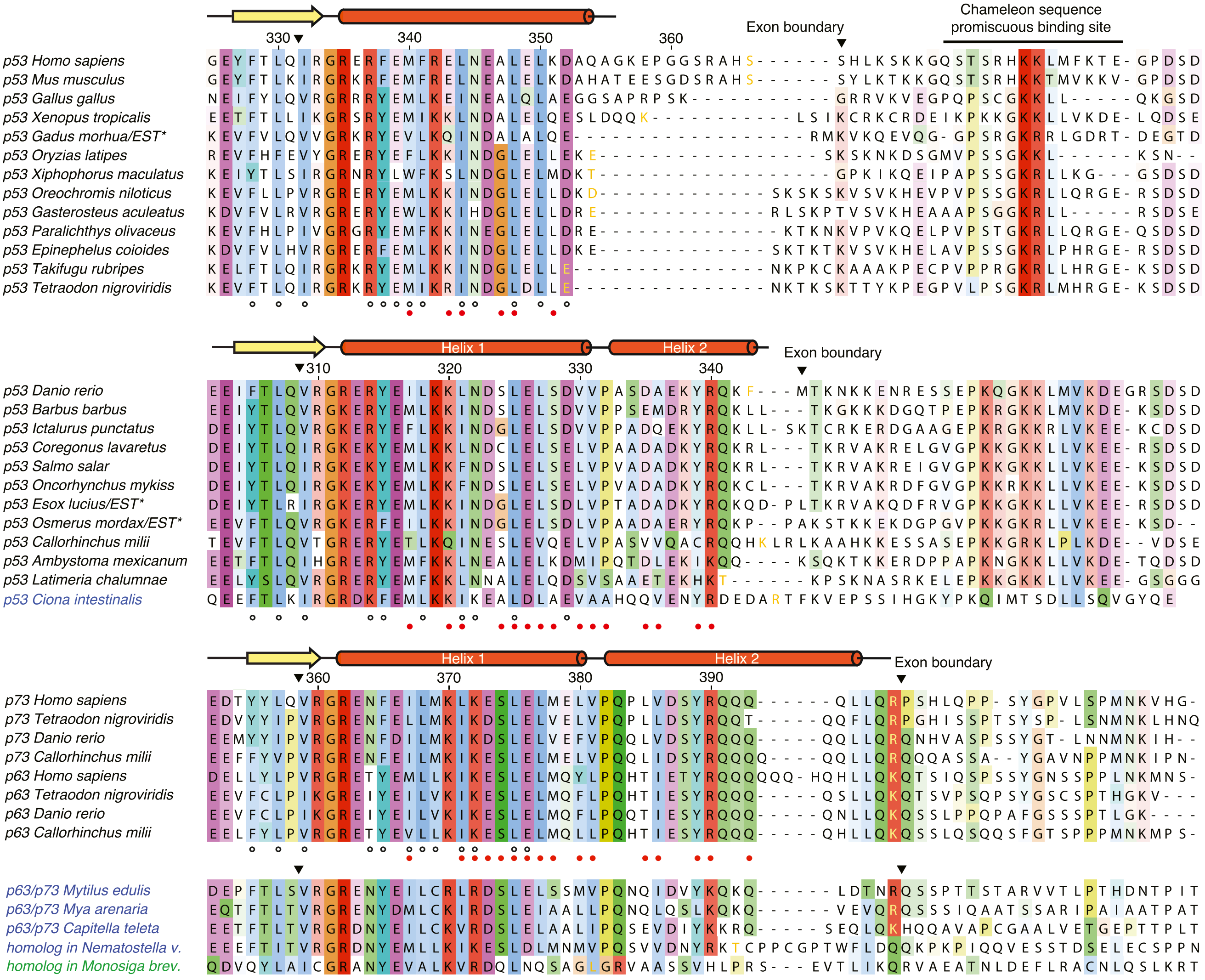

Figure 2. Sequence Alignment of p53 Family Oligomerization Domains

Sequences retrieved from the *expressed sequence tags* (ESTs) database are marked with asterisks. Amino acid residues are colored according to the Clustal color scheme based on sequence conservation and similarity. Secondary structure elements of human p53, zebrafish p53, and human p73 are shown above the corresponding alignments. The numbering of zebrafish p53 residues here and in the text is based on UniProt/TrEMBL entry G1K2L5 (length of 374 amino acids). Black inverted triangles indicate the position of introns. The last residue fully encoded by exon 9/10 is colored in light orange for sequences with known exon/intron structure. Black open circles below the alignments denote side chains that form subunit contacts stabilizing the dimeric interface (primary dimer), whereas red closed circles denote contact residues stabilizing the tetrameric interface (dimer-dimer contacts). The C-terminal region of p53 contains a chameleon sequence, a promiscuous binding site that can adopt different conformations depending on its interaction partner (Joerger and Fersht, 2010; Oldfield et al., 2008). Names of invertebrate species are highlighted in blue and that of the unicellular choanoflagellate *Monosiga brevicollis* in green. Accession codes, secondary structure predictions, and classification of p53 family sequences are given in Tables S1–S3.

including zebrafish (*Danio rerio*), barbel (*Barbus barbus*), Channel catfish (*Ictalurus punctatus*), Atlantic salmon (*Salmo salar*), rainbow trout (*Oncorhynchus mykiss*), Northern pike (*Esox lucius*), rainbow smelt (*Osmerus mordax*), and the spotted gar (*Lepisosteus oculatus*), a primitive fresh water fish. As in the case of the elephant shark, the corresponding regions have significant helical propensity (Table S1). In contrast, the p63/p73-like helix is clearly absent in medaka (*Oryzias latipes*), puffer fish (*Tetraodon nigroviridis*), tilapia (*Oreochromis niloticus*), stickleback (*Gasterosteus aculeatus*), and Atlantic cod (*Gadus morhua*). The loss of the second helix in these species appears to be the result of a significant deletion at the end of exon 9 (corresponding to exon 10 in the human homologs), rather than a series of missense mutations (Figure 2). The lobe-finned West Indian Ocean coelacanth (*Latimeria chalumnae*), together with lungfishes, one of the extant fish species most closely related to land vertebrates (Johanson et al., 2006), lacks some key residues of the second helix, but overall, the corresponding region has significant sequence similarity with the fishes possessing a putative second helix. In amphibians, the N-terminal cap region of the second helix is conserved in the neotenic salamanders, axolotl (*Ambystoma mexicanum*) and newt, but has significantly

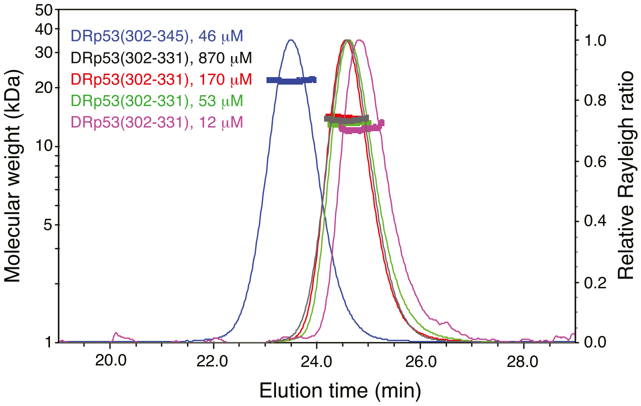

Figure 3. SEC-MALS Analysis of the Oligomerization State of Zebrafish p53 Tetramerization Domain Variants

Light-scattering curves for DRp53(302–331) and DRp53(302–345) are shown, with the measured protein concentration at the peak maximum given for each curve (monomer concentration). The curves were normalized to give the same peak heights. Calculated molar masses in the peak areas are shown as a thick line. For all three DRp53(302–345) concentrations measured (185 μM, 46 μM, and 9 μM), the calculated molecular weight was in excellent agreement with the theoretical molecular weight of the tetramers of 21.9 kDa, which includes an N-terminal GGS tag that was introduced as a result of the cloning strategy (e.g., 21.8 kDa measured for the shown curve at 46 μM). For high concentrations of DRp53(302–331), they were also in good agreement with the theoretical molecular weight of a tetramer of 15.2 kDa: 14.1 kDa at 170 μM and 13.9 kDa at 870 μM. At lower concentrations, the apparent molar mass of DRp53(302–331) decreased to 13.2 kDa (53 μM) and 12.4 kDa (12 μM), concomitant with an increase in the retention time, suggesting partial dissociation of the tetramers at low micromolar concentrations.

diverged in clawed frogs of the genus *Xenopus.* The latter also have a deletion at the end of the corresponding exon. The glutamine-rich region found at the end of helix 2 in p63 and p73 is more or less absent in the p53 sequences with a putative second helix, and only one or two glutamines remain.

The region that forms the tetramerization domain in human p53 is highly conserved among all three p53 family members (Figure 2). There are some variations of residues forming subunit contacts, providing an explanation as to why human p53, in contrast to human p63 and p73, forms stable tetramers without requiring a second helix. Most notably, human p53 has an intermolecular salt bridge formed by Arg337 and Asp352 that stabilizes the primary dimer (Figure S1). This salt bridge is conserved in the majority of vertebrate p53 sequences but is absent in p63 and p73. The p53 sequences of salmonidae have a lysine-glutamate pair in its place that may play a similar structural role. In addition, Asn345, which forms a hydrogen bond with a backbone oxygen from a neighboring subunit, is highly conserved in p53 across vertebrate species but is replaced by a lysine in p63 and p73. When analyzing the hydrophobic contact residues, there is no clear separation between p53 and p63/p73-like contact residues, and variations between the mammalian paralogs are often also found when comparing p53 orthologs (Figure 2).

Tracing the Ancestral Oligomerization Domain in Invertebrates

Our analysis of invertebrate p53 family genes showed a more diverse spectrum of oligomerization domain motifs, yet in many invertebrate species, an extended p63/p73-like domain was found, with a varying degree of conservation of the second helix (Figure 2). The extended p63/p73-like tetramerization domain motif is, for example, highly conserved in the single p53 family gene found in annelid worms (*Capitella teleta*) and molluscs (*Mya arenaria* and *Mytilus edulis*). The p53 homologs in *Drosophila* and *C. elegans* have unique oligomerization domains, and structural studies on the *C. elegans* domain demonstrated a potential evolutionary pathway from functional dimers to tetramers (Ou et al., 2007). Intriguingly, the tetramerization domain of a p53 family homolog found in the starlet sea anemone (*Nematostella vectensis*) is remarkably similar to that of human p63/p73, except for the missing glutamine-rich region (Figure 2). Unless an essentially identical domain has evolved independently twice, this traces the ancestral domain back to early metazoans before the divergence of Cnidaria and Bilateria. This also implies that the oligomerization domains of flies and even more so nematodes, which have an accelerated mutation rate (Cutter, 2010), have either significantly diverged from the primordial domain or evolved independently. They may therefore not necessarily resemble the ancestral domains of vertebrate p53 family proteins. It is, however, likely that the ancestral domain evolved from primordial dimers, as it has been shown that evolutionary pathways of homomeric proteins can be inferred from their symmetry and assembly pathways in solution (Levy et al., 2008). Divergent evolution of the *Drosophila* and *C. elegans* p53 homologs is also accompanied by differences in their regulatory pathways, most notably the absence of MDM2 genes in these genomes (Lane et al., 2010). The genome of the unicellular choanoflagellate *Monosiga brevcollis*, one of the closest living relatives of metazoans, contains two p53 family genes (King et al., 2008). Our analysis of these genes showed that one of the predicted p53 family proteins has a p63/p73-like oligomerization domain that lacks the second helix and a usually conserved leucine in the C-terminal region of helix 1 that makes intersubunit contacts in vertebrate p53 family members (Figure 2). This shortened oligomerization domain motif is closely followed by an extended, most likely structured region with homology to SAM-like domains that may play a stabilizing role, reminiscent of the scenario in the *C. elegans* homolog. In vertebrate and mollusk p63/p73 proteins, these domains are typically separated by about 90–100 residues, whereas in the *Trichoplax* p53 homolog the predicted oligomerization and SAM-like domains are located closer together.

Structure of the Zebrafish p53 Tetramerization Domain

To establish the role of the putative second helix in the p53 tetramerization domain found in many fish species, we performed crystallographic studies on zebrafish p53. Size-exclusion chromatography multiangle static light scattering (SEC-MALS) showed that residues 302–345 form stable tetramers (Figure 3). We then determined the X-ray structure of this domain in two different crystal forms at 1.97 Å and 2.2 Å resolution (Table 1). Crystal form I belongs to space group $C222_1$ and contains six chains in the asymmetric unit: one tetramer and one dimer that forms the same type of tetramer upon applying the crystallographic symmetry. Crystal form II belongs to space group $P2_1$ and contains three tetramers in the asymmetric unit. Overall, the zebrafish p53 tetramerization domain adopts the

Table 1. X-Ray Data Collection and Refinement Statistics

	DRp53(302–346)		DRp53(302–331)		
	(I)	(II)	(I)	(II)	(III)
Data Collection					
Space Group	$C222_1$	$P2_1$	$P2_12_12_1$	$P3_121$	$C2$
a (Å)	87.85	70.86	33.23	57.53	71.73
b (Å)	120.61	74.51	33.69	57.53	45.72
c (Å)	61.03	74.95	101.81	96.88	55.49
α (°)	90.00	90.00	90.00	90.00	90.00
β (°)	90.00	117.79	90.00	90.00	92.60
γ (°)	90.00	90.00	90.00	120.00	90.00
Molecules/AU	6	12	4	4	6
Resolution (Å)[a]	28.5–1.97 (2.08–1.97)	28.9–2.20 (2.32–2.20)	28.1–1.02 (1.08–1.02)	28.8–1.53 (1.61–1.53)	27.7–1.10 (1.16–1.10)
Unique reflections	23,226	34,925	59,076	28,720	68,831
Completeness (%)[a]	99.6 (99.3)	99.3 (99.5)	99.8 (99.9)	100 (100)	94.8 (87.4)
Multiplicity[a]	5.5 (5.5)	3.4 (3.3)	5.2 (5.0)	8.0 (7.4)	3.5 (3.3)
R_{merge} (%)[a]	4.7 (65.1)	4.4 (51.6)	3.4 (54.3)	3.5 (52.8)	6.2 (17.0)
$<I/\sigma_I>$[a]	18.1 (2.8)	14.8 (2.5)	19.3 (3.0)	25.8 (3.5)	12.1 (6.5)
Wilson B value (Å^2)	44.1	43.0	9.4	24.3	8.4
Refinement					
R_{cryst}, (%)[b]	21.7	22.8	15.8	14.8	14.3
R_{free}, (%)[b]	24.7	28.0	17.8	18.4	16.6
No. of atoms					
Protein[c]	1919	3919	1020	1005	1606
Water	58	59	121	72	140
Ions/additives	2	2	–	7	21
RMSD bonds (Å)	0.011	0.011	0.009	0.008	0.008
RMSD angles (°)	1.3	1.3	1.4	1.1	1.6
Mean B (Å^2)	55.2	57.8	15.5	32.9	12.9
PDB entry	4D1L	4D1M	4CZ5	4CZ6	4CZ7

[a]Values in parentheses are for the highest resolution shell.
[b]R_{cryst} and $R_{free} = \sum||F_{obs}| - |F_{calc}||/\sum|F_{obs}|$, where R_{free} was calculated with 5% of the reflections chosen at random and not used in the refinement.
[c]Number includes alternative conformations.

canonical tetramer structure of human p53, which is composed of a dimer of primary dimers with approximate D_2 symmetry, but importantly, it has an additional helix that engages in subunit contacts, reminiscent of p63 and p73 (Figure 1D). Individual subunits within the tetramer adopt a z-shaped conformation, consisting of a short β strand followed by two α helices (H1 and H2). The sharp turn between the β strand and helix H1 is facilitated by a strictly conserved glycine (Gly311), whereas the turn between the two helices is stabilized by a proline (Pro332) at the N-terminal cap of helix H2, which is strictly conserved in p63 and p73. Primary dimers are formed via an intermolecular β sheet and antiparallel helix packing, and two such dimers then form a tetramer via their helix interfaces, resulting in approximately orthogonal packing (Figure 1D). Key intersubunit contacts in the canonical region are conserved, most notably the salt bridge between Arg314 (Arg337 in human) and Asp329 (Asp352 in human) that stabilizes the primary dimer (Figure S1). The conserved leucines Leu321 (Leu344 in human) and Leu325 (Leu348 in human) play the same roles as in the human structure and stabilize the hydrophobic tetrameric and dimeric interfaces. There are some variations of hydrophobic residues at the center of the interface area. Instead of the phenylalanine in human p53 (Phe341), zebrafish p53, like human p63 and p73, has a smaller leucine residue (Leu318), which results in closer approach of adjacent H1 helices within the primary dimer (Figure S1).

Depending on the mobility of the C termini within the crystal lattice, we were able to build the model of helix H2 starting at Pro332 up to Met344. This helix reaches across the adjacent primary dimer within the tetramer, resulting in antiparallel packing of two H2 helices. Tyr339 plays a key role in stabilizing the H2-mediated subunit interface. It interacts with hydrophobic residues from a neighboring subunit and forms two intersubunit hydrogen bonds with Asp335 and Tyr339 (Figure 4A), although the latter is rather weak. Arg340 engages in intersubunit contacts with the backbone oxygen of Asp329 and the carboxylate group of Glu326, which is part of a larger salt-bridge cluster. This H2-mediated interaction network is, however, very different from that observed in human p63 and p73. The H2 helix is tilted by approximately 50° relative to the orientation found in p73, with the conserved proline at the N-terminal cap acting as a hinge

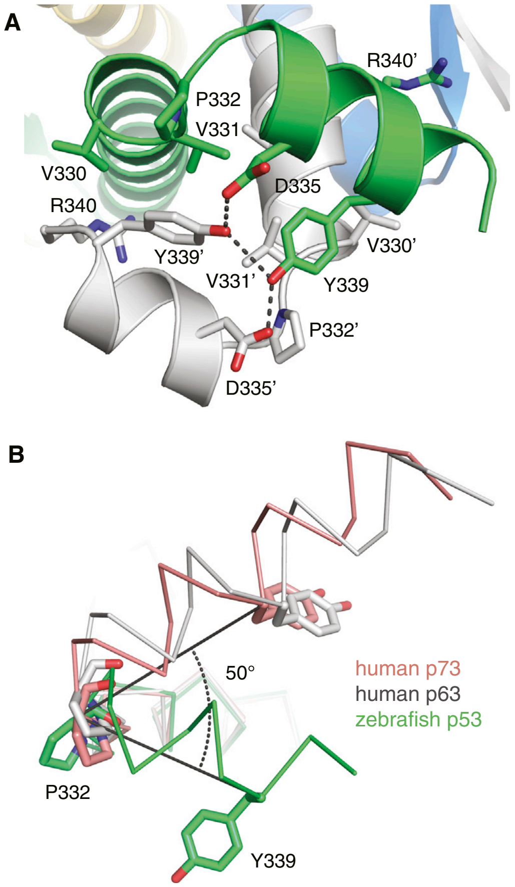

Figure 4. H2 Helix-Mediated Subunit Contacts in the Tetramerization Domain of Zebrafish p53

(A) Symmetrical packing of two adjacent H2 helices in the zebrafish p53 tetramer, highlighting the interaction network of Tyr339. Individual chains are shown as cartoons in different colors. Key residues of the H2 interface are shown as stick models, and intermolecular hydrogen bonds are indicated as broken lines.

(B) Superposition of tetramerization domain monomers of human p73 (salmon; PDB entry 2WQI), human p63 (gray; PDB entry 4A9Z) and zebrafish p53 (green), showing a 50° rotation of the H2 helix in the zebrafish p53 tetramer relative to its orientation in p73.

(Figure 4B). Because of the additional H2 interactions, the total surface area buried within the zebrafish tetramer is on average about 20% larger than in the human p53 tetramer (8,100 $Å^2$ versus 6,720 $Å^2$), which is also reflected in the dissociation constants, K_Ds, of the full-length protein tetramers (K_D = 3.6 nM for zebrafish p53 versus K_D = 19 nM for human p53 [Brandt et al., 2009)].

Conserved Tetramer Assembly in Zebrafish p53 in the Absence of Helix H2

Deletion of the C-terminal helix in p63 and p73 drastically reduces the stability of the tetramer and results in different packing of the primary dimers in higher order oligomers (Joerger et al., 2009; Natan and Joerger, 2012). To see whether the C-terminal helix of the zebrafish p53 tetramerization domain plays an equally pivotal role in stabilizing the overall architecture of the tetramer, we created a deletion variant comprising residues 302-331, DRp53(302-331). SEC-MALS showed that the truncated domain remained tetrameric at high micromolar concentrations, with the tetramers partially dissociating at low micromolar concentration (Figure 3). We determined its structure in three different crystal forms grown at different pH (5.0, 7.0, and 9.0) and ionic strength, up to a resolution of 1.0 Å (Table 1). In all three structures, DRp53(302–331) forms essentially identical tetramers with orthogonal packing of primary dimers as observed for the full-length tetramerization domain (Figures 5 and S2). The last turn of helix H1 partially unwinds in the truncated domain, and in some chains, the C-terminal carboxylate group of Val331 folds back onto the tetramer and forms a salt bridge with Arg314 from an adjacent subunit, thereby displacing Asp229 that normally interacts with the arginine (Figure 5C). Analysis of the salt-bridge patterns in different tetramers further revealed that Glu326 (Glu349 in human) can adopt alternative conformations and in some chains interacts directly with the guanidinium group of Arg314. This observation of fluctuating salt-bridge partners in the truncated zebrafish domain supports molecular dynamics simulations on the human protein, suggesting that the stabilizing Arg337-Asp352 salt bridge is part of a larger fluid salt-bridging cluster that includes Glu349 (Lwin et al., 2007). Taken together, the crystal structures of the truncated domain show that, although the second helix forms additional intersubunit contacts that stabilize the tetramer, it is not essential to the overall assembly geometry, which is stable across a broad range of pH and ionic strength.

The Role of Interface Coupling in Facilitating Domain Compaction

Intriguingly, systematic sequence analysis to search for substitutions that may have been responsible for stabilizing the shorter canonical p53 tetramer (compared to the equivalent region in p63 and p73) points to substitutions generating or modulating polar contacts within the primary dimer, whereas there are no obvious p53-specific variations at the dimer-dimer interface (Figure 2). This observation suggests that stabilization of the tetramer core is achieved through coupling of the dimer and tetramer interfaces. Crystal structures of the p63 and p73 tetramerization domains with truncated H2 helix reveal a notable difference in the packing angles of the H1 helices within the primary dimers compared to the full-length domains, with crucial consequences for the geometry and stability of higher order oligomers (Joerger et al., 2009; Natan and Joerger, 2012). In contrast, the packing angle of the H1 helices in zebrafish p53 is not significantly affected by deletion of the second helix, and the truncated domain has essentially the same dimer and tetramer architecture as human p53 (Figures 5B and 5D). The additional polar interactions in modern-day vertebrate p53, including the Arg337-Glu352 salt bridge (human numbering), therefore appear to lock the primary dimer in a geometry that provides a self-complementary surface poised for tetramer formation (Figure 5D). The most frequent human p53 germline cancer mutation, R337H, disrupts this intersubunit salt bridge and destabilizes the tetramer in a pH-dependent manner (DiGiammarino et al., 2002). The crucial role of interface coupling in the evolution of tetramers from dimers has recently been demonstrated for a number of protein families where changes that affect intersubunit geometry can be as important as mutations at the center of the tetrameric interface (Perica et al., 2012).

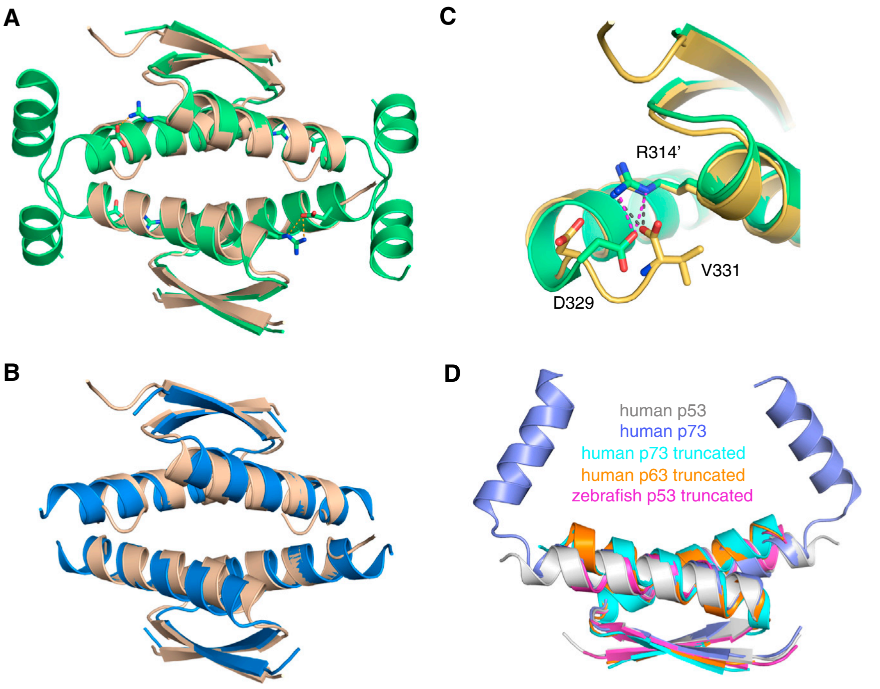

Figure 5. Conserved Tetramer Assembly of Zebrafish p53 Tetramerization Domain upon Deletion of Helix H2

(A) Superposition of the truncated domain lacking helix H2, DRp53(302–331) (light brown), onto the full-length domain with intact H2 helix, DRp53(302–346) (green), showing that the second helix is not essential to the overall architecture of the tetramer. The conserved Arg-Asp salt bridge stabilizing the primary dimers is shown as a stick model.

(B) Superposition of human (blue; PDB entry 1C26) and truncated zebrafish (light brown) p53 tetramerization domains.

(C) Alternative intradimer salt bridges in the truncated zebrafish p53 tetramerization domain lacking helix H2. In some chains of the truncated structure, the carboxy terminus of Val331 forms a salt bridge with Arg314 from an adjacent subunit within a primary dimer, thereby displacing the side chain of Asp329 that normally forms a salt bridge with the arginine. Alternative salt-bridge patterns were observed in all three crystal forms. The example shown is that of chains C and D in crystal form I (yellow) superimposed onto chains C and D in crystal form II (green).

(D) Packing of H1 helices in primary dimers of different p53 family oligomerization domains, showing changes in the packing of H1 helices in p63 and p73 domains upon deletion of the second helix. For clarity, only primary dimers are shown: human p53 TET domain (gray, PDB entry 1C26), truncated zebrafish p53 TET domain (magenta, PDB entry 4CZ5), human p73 TET domain (blue, PDB entry 2WQI), truncated human p73 TET domain (cyan, PDB entry 2WQJ), and truncated human p63 TET domain (orange, PDB entry 3ZY0).

See also Figure S2.

Independent Loss of the Second Helix in the p53 Tetramerization Domain at Various Stages of Vertebrate Evolution

Our sequence analyses suggest that the absence of a stabilizing second helix in the p53 tetramerization domain of mammals, clawed frogs, and the majority of bony fishes is the result of separate evolutionary processes (Figure 6). All fish species with a deletion of the second, p63/p73-like helix in their p53 tetramerization domain belong to the Acanthomorpha (spiny-rayed fishes), the crown group of teleost fishes that comprises nearly one-third of all living vertebrate species (Near et al., 2013; Nelson, 2006). Acanthomorph fishes can be found in virtually all known aquatic habitats from tropical coral reefs to Antarctic waters, mountain lakes, and the abyss of the ocean (Near et al., 2013; Nelson, 2006). The second helix was missing in all currently available acanthomorph p53 sequences, including species such as pufferfish and cod that diverged early in acanthomorph evolution. Recently published time-calibrated actinopterygian phylogenies, based on different but overlapping sets of nuclear genes and fossil age constraints, place the most recent common ancestor of the Acanthomorpha in the late Jurassic, about 145–165 million years ago (Betancur et al., 2013; Broughton et al., 2013; Near et al., 2012, 2013). Among the fish species with an extended p53 tetramerization domain motif, the Protacanthopterygii, which includes salmons, trouts, pikes (Esociformes), and smelts (Osmeriformes), are most closely related to Acanthomorpha. On the basis of the above timelines, the most recent common ancestor of Protacanthopterygii and Acanthomorpha lived at the end of the Triassic period about 210 million years ago (Betancur et al., 2013; Broughton et al., 2013; Near et al., 2012). These data suggest that the deletion(s) at the end of exon 9 leading to a more compact tetramerization domain occurred relatively early in the diversification of neoteleosts during the Jurassic (Figure 6). In amphibians, we found a similar, albeit smaller, deletion in the p53 protein of clawed frogs but not in axolotl and newts, which are thought to have a more slowly evolving p53 protein that is more closely related to the ancestral protein of tetrapods (Villiard et al., 2007). This deletion therefore most likely occurred after the Anura/Caudata divergence, the time point of which is currently still a matter of debate (Pyron, 2011; San Mauro, 2010). Sequence data from additional fish and amphibian species and reconciliation of molecular and fossil data should, in the future, permit more precise time estimates for the deletion events.

Functional Implications of Domain Compaction and Order-to-Disorder Transition in Human p53

In human p53, the region corresponding to the second helix in the tetramerization domain features several glycine and serine residues that provide flexibility. It is followed by the C-terminal regulatory domain, which is the site of numerous posttranslational modifications (Dai and Gu, 2010; Meek and Anderson, 2009) and has a unique binding promiscuity (Joerger and Fersht, 2010; Oldfield et al., 2008). The sequence alignment in Figure 2 suggests that this region evolved early in vertebrate evolution, which is probably directly linked to p53 acquiring novel somatic functions through rewiring of its signaling pathways. Compaction of the tetramerization domain and introduction of flexibility and adaptability in the flanking region may have freed up the C-terminal region to interact more efficiently with regulatory

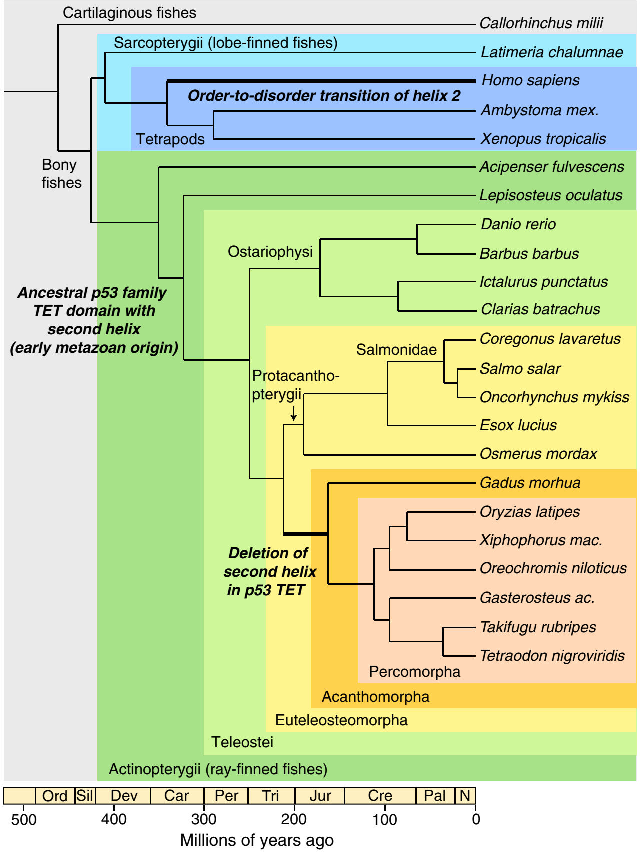

Figure 6. Time-Calibrated Phylogenetic Tree of Fishes and the Evolution of the p53 Tetramerization Domain

The tree was assembled based on the most recent fish phylogenies (Betancur et al., 2013; Near et al., 2012, 2013). Branch lengths reflect evolutionary distance in millions of years. Divergence times were either taken directly or extrapolated from Betancur et al. (2013) and the associated online tree (http://www.deepfin.org/OneZoomFish/fish.htm), except for the divergence of salamanders and clawed frogs which is taken from Pyron (2011). The corresponding geological periods are shown below the tree (Ord, Ordovician; Sil, Silurian, Dev, Devonian; Car, Carboniferous; Per, Permian; Tri, Triassic; Jur, Jurassic; Cre, Cretaceous; Pal, Paleogene; N, Neogene). p53 sequences are available for all species shown except for *Acipenser*. Vertebrate p53 tetramerization domains evolved from an ancestral, p63/p73-like domain with a second helix. Cartilaginous and many ray-finned fishes are predicted to have retained an extended domain with the ancestral feature of a second helix. All fish species with a shorter p53 tetramerization domain lacking helix H2 as a result of a deletion (see Figure 2 and Table S1) belong to the Acanthomorpha. In humans, the second helix in the p53 tetramerization domain has been lost independently through a series of point mutations, resulting in order-to-disorder transition.

proteins. As such, conversion of a structured helical segment into a region with a high degree of disorder may have contributed to the expansion and fine-tuning of the p53 interactome in higher vertebrates. In the case of the fish species without a second helix, the scenario is different. The loss of the helix is more likely the result of a substantial deletion rather than a series of point mutations that generated a potentially advantageous flexible linker region. It may therefore not necessarily be associated with an evolutionary advantage but represent the mere loss of a structural element that had become redundant. This notion is supported by our structural studies on zebrafish p53, showing that a truncated variant without the second helix forms tetramers with conserved interaction geometry. The structure of the zebrafish p53 tetramerization domain may therefore resemble a potential intermediate on the evolutionary pathway toward a more compact domain that had already acquired stabilizing mutations in the core structure of the tetramer, thus making the second helix dispensable and facilitating its subsequent deletion or order-to-disorder transition. The increased degree of disorder is a hallmark of cell-signaling and cancer-associated proteins, and in general, there is a weak correlation between the frequency of disorder and the complexity of an organism (Uversky et al., 2014). About 75% of mammalian signaling proteins are predicted to contain long regions of intrinsic disorder, resulting in enhanced signaling diversity (Dunker et al., 2008).

Conclusions

On the basis of the available genomic and structural data, the following scenario for the evolution of p53 tetramers emerges. The ancestral p63/p73-like protein that formed the last common ancestor of human p53, p63, and p73 had a second helix in its oligomerization domain that was essential for the formation of stable tetramers. This domain architecture was already established in some early metazoans and may have evolved via self-association of primordial dimers, as reflected in the symmetry and assembly pathways of present-day tetramers. After two gene duplication events that most likely occurred at the beginning of vertebrate evolution (Lane et al., 2011; Rutkowski et al., 2010), the second helix remained an integral part of the p63 and p73 tetramerization domains found in modern-day vertebrates (Coutandin et al., 2009; Joerger et al., 2009; Natan and Joerger, 2012). The p53 protein, however, diverged significantly during vertebrate radiation, resulting in independent loss of the second helix in tetrapods and spiny-rayed fishes (while other fishes retained this ancestral structural feature). At that stage, the core region of the p53 tetramerization domain had probably already acquired compensatory stabilizing mutations, such as the highly conserved intermolecular salt bridge within the primary dimer, making the additional helix redundant and allowing its deletion or gradual transformation without loss of fitness. Compaction of the domain in mammalian p53 generated a dynamic linker between the tetramerization domain and the C-terminal molecular recognition features, which may have contributed to binding diversity and expansion of p53's regulatory functions.

EXPERIMENTAL PROCEDURES

Sequence Analyses

Sequences of p53 family members were collected using UniProt (http://www.uniprot.org), the NCBI server (http://www.ncbi.nlm.nih.gov/), and the ENSEMBL genome browser (http://www.ensembl.org). Multiple p53 family sequences were then used as queries in a TBLASTN (Altschul et al., 1997) search against the nucleotide and *expressed sequence tags* databases at NCBI. Accession codes and classification of p53 family sequences are given in Tables S2 and S3. Sequences were aligned using MUSCLE (Edgar, 2004) and visualized and edited manually using Jalview (Waterhouse et al., 2009). Consensus secondary structure predictions were performed using the NPS@ web server (Combet et al., 2000).

Cloning, Gene Expression, and Protein Purification

cDNA of zebrafish p53 was purchased from Source BioScience (UK). Different regions of the tetramerization domain were amplified by PCR and cloned into a modified pRSET vector (pRSET-HLTEV, courtesy of Dr. Mark Allen, MRC Laboratory of Molecular Biology, Cambridge) using BamHI and EcoRI restriction sites. The resulting plasmids encode a fusion protein with an N-terminal 6xHis tag, followed by the lipoyl domain of the dihydrolipoamide acetyltransferase from *Bacillus stearothermophilus* and a TEV-protease cleavage site followed by the p53 tetramerization domain variant. The recombinant proteins were produced in *Escherichia coli* strain C41 and purified using a Ni-affinity column followed by cleavage with TEV protease overnight at 4°C. The protein was then further purified via a second Ni-affinity column and gel filtration on a Superdex 75 Prep Grade column (final buffer: 20 mM Tris [pH 7.4], 50 mM NaCl, 5 mM DTT). Purified samples were concentrated to 12–17 mg/ml, flash frozen in liquid nitrogen, and stored at −80°C.

SEC-MALS

SEC-MALS measurements were performed at room temperature using a Wyatt Heleos II 18 angle light scattering instrument coupled to a Wyatt Optilab rEX online refractive index detector. Samples of DRp53(302-331) and DRp53(302-345) in 50 mM Tris (pH 7.5) and 150 mM NaCl were resolved on a Superdex 75 10/300 analytical gel filtration column at a flow rate of 0.5 ml/min and passed through the light-scattering and refractive index detectors in a standard SEC-MALS format. Data analysis was performed using the ASTRA5 software (Wyatt Technology), with bovine serum albumin as a calibration standard.

Crystallization and Structure Determination

Crystals of DRp53(302-346) and DRp53(302-331) were grown at 20°C by sitting-drop vapor diffusion whereby 500 nL protein solution (12–17 mg/ml in 20 mM Tris [pH 7.4], 50 mM NaCl, and 5 mM DTT) and 500 nL crystallization buffer were mixed above a reservoir of 100 μl crystallization buffer. Two different crystal forms grew for DRp53(302–346) within a few days: crystal form I grew with a crystallization buffer of 50 mM zinc acetate and 20% (w/v) polyethylene glycol 3,350, and crystal form II with 160 mM zinc acetate, 80 mM sodium cacodylate (pH 6.5), 12% (w/v) polyethylene glycol 8,000, and 19% (w/v) glycerol. Crystal form I was soaked in mother liquor complemented with 20% glycerol before flash freezing in liquid nitrogen. Crystal form II was flash frozen directly from the crystallization drop. For DRp53(302–331) three well-diffracting crystal forms were obtained using the following reservoir solutions: crystal form I: 2.4 M ammonium sulfate, 100 mM bicine (pH 9.0); crystal form II: 50% (w/v) polyethylene glycol 200, 100 mM Tris (pH 7.0); crystal form III: 1.8 M sodium/potassium phosphate (pH 5.0). Before flash freezing in liquid nitrogen, crystal form I was soaked in mother liquor complemented with 22% glucose and crystal form III in mother liquor complemented with 20% glycerol. The crystallization buffer of crystal form II was already cryogenic, and the crystals were flash frozen directly from the crystallization drops. X-ray data sets were collected at 100 K at the Diamond Light Source (beamline I03) (Oxford). The data were integrated with XDS (Kabsch, 2010) and scaled with SCALA (Evans, 2006). Phases for DRp53(302–346) crystal form I were calculated by molecular replacement in PHASER (McCoy et al., 2007) using the primary dimer of the human tetramerization domain (PDB entry 1C26) (Jeffrey et al., 1995) as a search model. The structure was refined by iterative cycles of manual model building in COOT (Emsley et al., 2010) and restrained refinement with PHENIX (Adams et al., 2002) and REFMAC5 (Murshudov et al., 2011). During later stages of refinement, translation/liberation/screw parameters were introduced. The model of the tetramer in crystal form I was then used for molecular replacement of crystal form II. The structure of the short variant, DRp53(302–331), in each of the three different crystal forms was solved by molecular replacement with a truncated primary dimer of the long variant as a search model. Refinement was performed using REFMAC5 with manual model building in COOT. Waters were built using ARP/wARP (Langer et al., 2008). The final models were validated using MolProbity (Davis et al., 2007). Data collection and refinement statistics are given in Table 1. Buried surface areas were calculated using the PISA server (Krissinel and Henrick, 2007), and structural figures were prepared by use of PyMOL (http://www.pymol.org).

ACCESSION NUMBERS

The atomic coordinates and structure factors of the zebrafish p53 tetramerization domain crystal structures have been deposited in the PDB, http://www.pdb.org (PDB ID codes 4CZ5, 4CZ6, 4CZ7, 4D1L, and 4D1M).

SUPPLEMENTAL INFORMATION

Supplemental Information includes two figures and three tables and can be found with article online at http://dx.doi.org/10.1016/j.str.2014.07.010.

AUTHOR CONTRIBUTIONS

A.C.J. and A.A. performed sequence and phylogenetic analyses, R.W. expressed and purified proteins, and A.C.J. designed the project, performed the structural studies, and wrote the manuscript.

ACKNOWLEDGMENTS

We thank Sir Alan Fersht for his continuing support and M. Madan Babu and Caroline Blair for valuable comments on the manuscript. We acknowledge Christopher M. Johnson for advice and helpful comments and for access to the MRC-LMB biophysics facility instrumentation. We also thank the staff at beamline I03 of the Diamond Light Source for technical support during X-ray data collection. This work was funded by Medical Research Council Programme Grant G0901534.

Received: May 9, 2014
Revised: July 24, 2014
Accepted: July 25, 2014
Published: September 2, 2014

REFERENCES

Adams, P.D., Grosse-Kunstleve, R.W., Hung, L.W., Ioerger, T.R., McCoy, A.J., Moriarty, N.W., Read, R.J., Sacchettini, J.C., Sauter, N.K., and Terwilliger, T.C. (2002). PHENIX: building new software for automated crystallographic structure determination. Acta Crystallogr. D Biol. Crystallogr. *58*, 1948–1954.

Altschul, S.F., Madden, T.L., Schäffer, A.A., Zhang, J., Zhang, Z., Miller, W., and Lipman, D.J. (1997). Gapped BLAST and PSI-BLAST: a new generation of protein database search programs. Nucleic Acids Res. *25*, 3389–3402.

Belyi, V.A., Ak, P., Markert, E., Wang, H., Hu, W., Puzio-Kuter, A., and Levine, A.J. (2010). The origins and evolution of the p53 family of genes. Cold Spring Harb. Perspect. Biol. *2*, a001198.

Betancur, R.R., Broughton, R.E., Wiley, E.O., Carpenter, K., López, J.A., Li, C., Holcroft, N.I., Arcila, D., Sanciangco, M., Cureton Ii, J.C., et al. (2013). The tree of life and a new classification of bony fishes. PLoS Curr. Published online April 18, 2013. http://dx.doi.org/10.1371/currents.tol.53ba26640df0ccaee75bb165c8c26288.

Brandt, T., Petrovich, M., Joerger, A.C., and Veprintsev, D.B. (2009). Conservation of DNA-binding specificity and oligomerisation properties within the p53 family. BMC Genomics *10*, 628.

Broughton, R.E., Betancur-R, R., Li, C., Arratia, G., and Ortí, G. (2013). Multi-locus phylogenetic analysis reveals the pattern and tempo of bony fish evolution. PLoS Curr. Published online April 16, 2013. http://dx.doi.org/10.1371/currents.tol.2ca8041495ffafd0c92756e75247483e.

Clore, G.M., Ernst, J., Clubb, R., Omichinski, J.G., Kennedy, W.M., Sakaguchi, K., Appella, E., and Gronenborn, A.M. (1995). Refined solution structure of the oligomerization domain of the tumour suppressor p53. Nat. Struct. Biol. *2*, 321–333.

Combet, C., Blanchet, C., Geourjon, C., and Deléage, G. (2000). NPS@: network protein sequence analysis. Trends Biochem. Sci. *25*, 147–150.

Coutandin, D., Löhr, F., Niesen, F.H., Ikeya, T., Weber, T.A., Schäfer, B., Zielonka, E.M., Bullock, A.N., Yang, A., Güntert, P., et al. (2009). Conformational stability and activity of p73 require a second helix in the tetramerization domain. Cell Death Differ. *16*, 1582–1589.

Cutter, A.D. (2010). Molecular evolution inferences from the C. elegans genome. WormBook: the online review of C. elegans biology, 1–14.

Dai, C., and Gu, W. (2010). p53 post-translational modification: deregulated in tumorigenesis. Trends Mol. Med. *16*, 528–536.

Davis, I.W., Leaver-Fay, A., Chen, V.B., Block, J.N., Kapral, G.J., Wang, X., Murray, L.W., Arendall, W.B., 3rd, Snoeyink, J., Richardson, J.S., and Richardson, D.C. (2007). MolProbity: all-atom contacts and structure validation for proteins and nucleic acids. Nucleic Acids Res. *35*, W375–W383.

Deutsch, G.B., Zielonka, E.M., Coutandin, D., Weber, T.A., Schäfer, B., Hannewald, J., Luh, L.M., Durst, F.G., Ibrahim, M., Hoffmann, J., et al. (2011). DNA damage in oocytes induces a switch of the quality control factor TAp63α from dimer to tetramer. Cell *144*, 566–576.

DiGiammarino, E.L., Lee, A.S., Cadwell, C., Zhang, W., Bothner, B., Ribeiro, R.C., Zambetti, G., and Kriwacki, R.W. (2002). A novel mechanism of tumorigenesis involving pH-dependent destabilization of a mutant p53 tetramer. Nat. Struct. Biol. *9*, 12–16.

Dötsch, V., Bernassola, F., Coutandin, D., Candi, E., and Melino, G. (2010). p63 and p73, the ancestors of p53. Cold Spring Harb. Perspect. Biol. *2*, a004887.

Dunker, A.K., Silman, I., Uversky, V.N., and Sussman, J.L. (2008). Function and structure of inherently disordered proteins. Curr. Opin. Struct. Biol. *18*, 756–764.

Edgar, R.C. (2004). MUSCLE: multiple sequence alignment with high accuracy and high throughput. Nucleic Acids Res. *32*, 1792–1797.

Emsley, P., Lohkamp, B., Scott, W.G., and Cowtan, K. (2010). Features and development of Coot. Acta Crystallogr. D Biol. Crystallogr. *66*, 486–501.

Evans, P. (2006). Scaling and assessment of data quality. Acta Crystallogr. D Biol. Crystallogr. *62*, 72–82.

Jeffrey, P.D., Gorina, S., and Pavletich, N.P. (1995). Crystal structure of the tetramerization domain of the p53 tumor suppressor at 1.7 angstroms. Science *267*, 1498–1502.

Joerger, A.C., and Fersht, A.R. (2008). Structural biology of the tumor suppressor p53. Annu. Rev. Biochem. *77*, 557–582.

Joerger, A.C., and Fersht, A.R. (2010). The tumor suppressor p53: from structures to drug discovery. Cold Spring Harb. Perspect. Biol. *2*, a000919.

Joerger, A.C., Rajagopalan, S., Natan, E., Veprintsev, D.B., Robinson, C.V., and Fersht, A.R. (2009). Structural evolution of p53, p63, and p73: implication for heterotetramer formation. Proc. Natl. Acad. Sci. USA *106*, 17705–17710.

Johanson, Z., Long, J.A., Talent, J.A., Janvier, P., and Warren, J.W. (2006). Oldest coelacanth, from the Early Devonian of Australia. Biol. Lett. *2*, 443–446.

Kabsch, W. (2010). Xds. Acta Crystallogr. D Biol. Crystallogr. *66*, 125–132.

King, N., Westbrook, M.J., Young, S.L., Kuo, A., Abedin, M., Chapman, J., Fairclough, S., Hellsten, U., Isogai, Y., Letunic, I., et al. (2008). The genome of the choanoflagellate Monosiga brevicollis and the origin of metazoans. Nature *451*, 783–788.

Krissinel, E., and Henrick, K. (2007). Inference of macromolecular assemblies from crystalline state. J. Mol. Biol. *372*, 774–797.

Lane, D.P. (1992). Cancer. p53, guardian of the genome. Nature *358*, 15–16.

Lane, D.P., Cheok, C.F., Brown, C., Madhumalar, A., Ghadessy, F.J., and Verma, C. (2010). Mdm2 and p53 are highly conserved from placozoans to man. Cell Cycle *9*, 540–547.

Lane, D.P., Madhumalar, A., Lee, A.P., Tay, B.H., Verma, C., Brenner, S., and Venkatesh, B. (2011). Conservation of all three p53 family members and Mdm2 and Mdm4 in the cartilaginous fish. Cell Cycle *10*, 4272–4279.

Langer, G., Cohen, S.X., Lamzin, V.S., and Perrakis, A. (2008). Automated macromolecular model building for X-ray crystallography using ARP/wARP version 7. Nat. Protoc. *3*, 1171–1179.

Lee, W., Harvey, T.S., Yin, Y., Yau, P., Litchfield, D., and Arrowsmith, C.H. (1994). Solution structure of the tetrameric minimum transforming domain of p53. Nat. Struct. Biol. *1*, 877–890.

Levine, A.J., Tomasini, R., McKeon, F.D., Mak, T.W., and Melino, G. (2011). The p53 family: guardians of maternal reproduction. Nat. Rev. Mol. Cell Biol. *12*, 259–265.

Levy, E.D., Boeri Erba, E., Robinson, C.V., and Teichmann, S.A. (2008). Assembly reflects evolution of protein complexes. Nature *453*, 1262–1265.

Luh, L.M., Kehrloesser, S., Deutsch, G.B., Gebel, J., Coutandin, D., Schäfer, B., Agostini, M., Melino, G., and Dötsch, V. (2013). Analysis of the oligomeric state and transactivation potential of TAp73α. Cell Death Differ. *20*, 1008–1016.

Lwin, T.Z., Durant, J.J., and Bashford, D. (2007). A fluid salt-bridging cluster and the stabilization of p53. J. Mol. Biol. *373*, 1334–1347.

McCoy, A.J., Grosse-Kunstleve, R.W., Adams, P.D., Winn, M.D., Storoni, L.C., and Read, R.J. (2007). Phaser crystallographic software. J. Appl. Cryst. *40*, 658–674.

Meek, D.W., and Anderson, C.W. (2009). Posttranslational modification of p53: cooperative integrators of function. Cold Spring Harb. Perspect. Biol. *1*, a000950.

Murshudov, G.N., Skubák, P., Lebedev, A.A., Pannu, N.S., Steiner, R.A., Nicholls, R.A., Winn, M.D., Long, F., and Vagin, A.A. (2011). REFMAC5 for the refinement of macromolecular crystal structures. Acta Crystallogr. D Biol. Crystallogr. *67*, 355–367.

Natan, E., and Joerger, A.C. (2012). Structure and kinetic stability of the p63 tetramerization domain. J. Mol. Biol. *415*, 503–513.

Near, T.J., Eytan, R.I., Dornburg, A., Kuhn, K.L., Moore, J.A., Davis, M.P., Wainwright, P.C., Friedman, M., and Smith, W.L. (2012). Resolution of ray-finned fish phylogeny and timing of diversification. Proc. Natl. Acad. Sci. USA *109*, 13698–13703.

Near, T.J., Dornburg, A., Eytan, R.I., Keck, B.P., Smith, W.L., Kuhn, K.L., Moore, J.A., Price, S.A., Burbrink, F.T., Friedman, M., and Wainwright, P.C. (2013). Phylogeny and tempo of diversification in the superradiation of spiny-rayed fishes. Proc. Natl. Acad. Sci. USA *110*, 12738–12743.

Nedelcu, A.M., and Tan, C. (2007). Early diversification and complex evolutionary history of the p53 tumor suppressor gene family. Dev. Genes Evol. *217*, 801–806.

Nelson, J.S. (2006). Fishes of the World, Fourth Edition. (New York: Wiley).

Oldfield, C.J., Meng, J., Yang, J.Y., Yang, M.Q., Uversky, V.N., and Dunker, A.K. (2008). Flexible nets: disorder and induced fit in the associations of p53 and 14-3-3 with their partners. BMC Genomics *9* (*Suppl 1*), S1.

Ou, H.D., Löhr, F., Vogel, V., Mäntele, W., and Dötsch, V. (2007). Structural evolution of C-terminal domains in the p53 family. EMBO J. *26*, 3463–3473.

Pankow, S., and Bamberger, C. (2007). The p53 tumor suppressor-like protein nvp63 mediates selective germ cell death in the sea anemone Nematostella vectensis. PLoS ONE *2*, e782.

Perica, T., Chothia, C., and Teichmann, S.A. (2012). Evolution of oligomeric state through geometric coupling of protein interfaces. Proc. Natl. Acad. Sci. USA *109*, 8127–8132.

Pyron, R.A. (2011). Divergence time estimation using fossils as terminal taxa and the origins of Lissamphibia. Syst. Biol. *60*, 466–481.

Rutkowski, R., Hofmann, K., and Gartner, A. (2010). Phylogeny and function of the invertebrate p53 superfamily. Cold Spring Harb. Perspect. Biol. *2*, a001131.

San Mauro, D. (2010). A multilocus timescale for the origin of extant amphibians. Mol. Phylogenet. Evol. *56*, 554–561.

Uversky, V.N., Davé, V., Iakoucheva, L.M., Malaney, P., Metallo, S.J., Pathak, R.R., and Joerger, A.C. (2014). Pathological unfoldomics of uncontrolled chaos: intrinsically disordered proteins and human diseases. Chem. Rev. *114*, 6844–6879.

Villiard, E., Brinkmann, H., Moiseeva, O., Mallette, F.A., Ferbeyre, G., and Roy, S. (2007). Urodele p53 tolerates amino acid changes found in p53 variants linked to human cancer. BMC Evol. Biol. *7*, 180.

Vousden, K.H., and Prives, C. (2009). Blinded by the Light: The Growing Complexity of p53. Cell *137*, 413–431.

Waterhouse, A.M., Procter, J.B., Martin, D.M., Clamp, M., and Barton, G.J. (2009). Jalview Version 2: a multiple sequence alignment editor and analysis workbench. Bioinformatics *25*, 1189–1191.

Xue, B., Brown, C.J., Dunker, A.K., and Uversky, V.N. (2013). Intrinsically disordered regions of p53 family are highly diversified in evolution. Biochim. Biophys. Acta *1834*, 725–738.

Structure, Volume *22*

Supplemental Information

Tracing the Evolution

# of the p53 Tetramerization Domain

Andreas C. Joerger, Rainer Wilcken, and Antonina Andreeva

Table S1, related to Figures 2 and 6.

Consensus secondary structure prediction of p53 C-terminal regions in vertebrates

Species	Consensus secondary structure prediction*
Human<br>*Homo sapiens*	GEYFTLQIRGRERFEMFRELNEALELKDAQ**A**GKEPGGSRAHSSHLKSKKGQSTSRHKKLMFKTEGPDSD<br>cceeeeeecchhhhhhhhhhhhhhhhhhhhccccccccccc?ccccccccccccchheeeeeccccccc
Dolphin<br>*Delphinapterus leucas*	GEYFTLQIRGRERFEMFRELNEALELKDAQ**A**GKEPGESRAHSSHLKSKKGQSPSRHKKLMFKREGPDSD<br>cceeeeeecchhhhhhhhhhhhhhhhhhhhhcccccccccccccccccccccccchhh??eeccccccc
Mouse<br>*Mus musculus*	GEYFTLKIRGRKRFEMFRELNEALELKDAH**A**TEESGDSRAHSSYLKTKKGQSTSRHKKTMVKKVGPDSD<br>cceeeeeeccchhhhhhhhhhhhhhhhhhhhhhcccccc?ch?hhcccccccccchhheeeeccccccc
Chicken<br>*Gallus gallus*	NEIFYLQVRGRRRYEMLKEINEALQLAEGG**S**APRPSKGRRVKVEGPQPSCGKKLLQKGSD<br>cceeeeeecc?hhhhhhhhhhhhhhhhcccccccccccceeee?cccccccc?hhccccc
African clawed frog<br>*Xenopus laevis*	EEIFTLRIKGRSRYEMIKKLNDALELQESL**D**QQKVTIKCRKCRDEIKPKKGKKLLVKDEQPDSE<br>c?eeeeeecccchhhhhhhhhhhhhhhhhhhhhh???e?h?ccccccccccceeeeeccccccc
Axolotl<br>*Ambystoma mexicanum*	EETFTLQIHGRERYEMFKKLNEALELKDMI**P**QTDLEKIKQQKSQKTKKERDPPAPKNGKKLLVKDETQ<br>cceeeeeeccchhhhhhhhhhhhhhhhhcccc?hhhhhhhhhh???ccccccccccccceeeeecccc
Coelacanth<br>*Latimeria chalumnae*	EELYSLQVRGRERYEMFKKLNNALELQDSV**S**AAETEKHKTKPSKNASRKELEPKKGKKLLVKEEGSGGG<br>c?h?eeeecc?hhhhhhhhh?hhhhhhhhhhhhhhhhcccccccch?hhhccccccceeeeeecccccc
Elephant shark<br>*Callorhinchus milli*	TEVFTLQVTGRERYETLKQINESLEVQELV**P**ASVVQACRQQHKLRLKAAHKKESSASEPKKGRKLPLKDEVDSE<br>ceeeeeeecc?hhhhhhhhhhhhhhhhhhcc?hhhhhhhhhhhhhhhhhhcccccccccccccccccccccccc
Spotted gar<br>*Lepisosteus oculatus*	EEIFTLQVRGRERFEMLKKINESLELKDLV**P**VADLEKYRQKLHTRSSSRREKDKEKEPKKGKKLLVKEEKTDSD<br>cceeeeeecc?hhhhhhhhhhhhhhh?ccccchhhhhhhhhhccccchhhh?cccccccccceeeeeecccccc
Zebrafish<br>*Danio rerio*	EEIFTLQVRGRERYEILKKLNDSLELSDVV**P**ASDAEKYRQKFMTKNKKENRESSEPKQGKKLMVKDEGRSDSD<br>c?eeeeeecc?hhhhhhhhhccccccccccccchhhhhhhhhhh?ccccccccccccccceeeeecccccccc
Barbel<br>*Barbus barbel*	EEIYTLQVRGKERYEMLKKINDSLELSDVV**P**PSEMDRYRQKLLTKGKKKDGQTPEPKRGKKLMVKDEKSDSD<br>cceeeeeecc?hhhhhhhhhhcc?h?ccccccc?hhhhhhhhhhcccccccccccccccceeeeeccccccc
Channel catfish<br>*Ictalurus punctatus*	DEIYTLQVRGKERYEFLKKINDGLELSDVV**P**PADQEKYRQKLLSKTCRKERDGAAGEPKRGKKRLVKEEKCDSD<br>cceeeeeecc?hhhhhhhhhcccccccccccccchhhhhhhhhhhhhh?hcccccccccccc?eeeeccccccc
Walking catfish<br>*Clarias batrachus*	EEIFLLQIRGRDRFNMLKTINDSLELMDMM**P**AADKDKYRQKRTLKNLKKRPGCGKKTAPKR<br>cceeeeeecccchhhhhhhhhhhhhhhhhcchhchhhhhhhhhhhhhcccccccccccccc
Atlantic salmon<br>*Salmo salar*	DEIYTLQIRGKEKYEMLKKFNDSLELSELV**P**VADADKYRQKRLTKRVAKREIGVGPKKGKKLLVKEEKSDSD<br>cceeeeeeccchhhhhhhhhcchhhhhh?ccchhhhhhhhhhhhhhhhhhhccccccccceeeeeecccccc
Rainbow trout<br>*Oncorhynchus mykiss*	DEIYTLQIRGKEKYEMLKKFNDSLELSELV**P**VADADKYRQKCLTKRVAKRDFGVGPKKRKKLLVKEEKSDSD<br>cceeeeeeccc?hhhhhhhhcchhhhhh?ccccc?hhhhhhhhhhhhh?ccccccccc?cheeeeccccccc
European whitefish<br>*Coregonus lavaretus*	DEIYTLQIRGKEKYEMLKKLNDCLELSELV**P**AADADKYRQKRLTKRVAKRELGVGPKKGKKLLVKEERSDSD<br>cceeeeeeccchhhhhhhhhhhhhhhhhhccc?hhhhhhhhhhhhhhhhhhccccccccceeeeeccccccc
Northern pike<br>*Esox lucius*	DEIYTLRIRGKERYEMLKKINDGLELSDLV**P**TADADKYRQKQDPLTKRVAKQDFRVGPKKGKKLLVKEERSDSD<br>cceeeeeecc?hhhhhhhhhccccc?cccccccchhhhhhccc?hhhhhhhhcccccccccceeeeeccccccc
Rainbow smelt<br>*Osmerus mordax*	EEVFTLQVRGKERFEILKKINDGLELSDLV**P**AADAERYRQKPPAKSTKKEKDGPGVPKKGKRLLVKEEKSD<br>cceeeeeccccchhhhhhhhccccchchhcchhhhhhhhcccccccccccccccccccccceeeeeccccc
Atlantic cod<br>*Gadus morhua*	KEVFVLQVVGRKRYEVLKQINDALALQERM**K**VKQEVQGGPSRGKRRLGDRTDEGTD<br>cceeeeee?cchhhhhhhhhhhhhhhhhhhhhhhhhcccccccccccccccccccc
Japanese rice fish<br>*Oryzias latipes*	REVFHFEVYGRERYEFLKKINDGLELLEKE**S**KSKNKDSGMVPSSGKKLKSN<br>cheeeeeeccchhhhhhhhhcchhhhhhhhccccccccccccccccccccc
Turquoise killifish<br>*Nothobranchius furzeri*	KEMIPLYIQGRKKWNLMKRISDGLDLVEKE**K**KQLLVQEVLPTSGKRLLKKDRSDSD<br>ccccceeecccchhhhhhhhhc?hhhhhhhhhhhhhhh?ccccchhhhhccccccc
Green swordtail<br>*Xiphophorus helleri*	KEIYTLSIRGRNRYLWFKSLNDGLELMDKT**G**PKIKQEIPAPSSGKRLLKGGSDSD<br>ceeeeeeecccc?eeeehhhcc?hhhhhccccccc?ccccccccc??eccccccc
Pufferfish<br>*Takifugu rubripes*	KELFTLQIRGRKRYEMLKKINDGLELLENK**P**KCKAAAKPECPVPPRGKRLLHRGEKSDSD<br>cc??ee??cchhhhhhhhhhcc?hhhhhccccccccccccccccccccceeecccccccc
Three-spined stickleback<br>*Gasterosteus aculeatus*	KDVFVLRVRGRERYEWLKKIHDGLELLDRE**R**LSKPTVSVKHEAAAPSGGKRLLRSDSE<br>cceeeeeeccchhhhhhhhhhchhhhhhhhcccccc?eeeeccccccccceeeecccc
Orange-spotted grouper<br>*Epinephelus coioides*	KDVFVLHVRGRERFEMLKKINDGLELLDKE**S**KTKTKVSVKHELAVPSSGKRLPHRGERSDSD<br>cceeeee?cc?hhhhhhhhhcc?hhhh?hcccccceeeeeeeeecccccccccccccccccc
Mangrove killifish<br>*Kryptolebias marmoratus*	KEVFTLHVRGRERYEMLKKINAGLELLDTD**G**KRTKQEKKFPKKDKSDSD<br>ceeeeeeecc?hhhhhhhhhhccc??hcccccccc??cccccccccccc
Climbing perch<br>*Anabas testudineus*	KDVFVLHVRGRDRFEMLKKINDGLELLDKK**N**KTKSKASTKHGVPVPCSGKRLLQRGDRSDSD<br>cceeeeeeccc?hhhhhhhhcc?hhhhhccccccccccccccccccccccc??ccccccccc
Olive flounder<br>*Paralichthys olivaceus*	KEVFHLPIVGRGRYEMFKKINEGLELLDRE**K**TKKVPVKQELPVPSTGKRLLQRGEQSDSD<br>ceeeeeecccc?hhhhhhhhhhhhhhhhhcccccccccccccccccc?hhh?cccccccc

*Consensus secondary structure prediction (DPM, DSC, GOR IV. HNN, PHD, Predator, SIMPA96, SOPM) using Network Protein Sequence Analysis (Combet *et al*., 2000). h = α-helix, e = β-strand, c = coil, ? = ambiguous. The residue corresponding to the N-terminal cap of helix H2 in the tetramerization domain of human p63 and p73 is in bold and underlined. Names of ray-finned fishes are highlighted in different colors: green, Acanthomorpha; blue, other ray-finned fishes.

Table S2, related to Figures 2 and 6.

Accession codes of selected vertebrate p53 family proteins

Species	Genome sequenced	Protein	Database	Accession code	Expression
Human	yes	p53	UniProt	P04637	yes
Homo sapiens		p63	UniProt	Q17RN8	yes
		p73	UniProt	Q9H3D4	yes
Mouse	yes	p53	UniProt	Q549C9	yes
Mus musculus		p63	UniProt	O88898	yes
		p73	UniProt	Q9JJP2	yes
Rabbit	yes	p53	UniProt	Q95330	yes
Oryctolagus cuniculus		p63	Ensembl	G1TBP7	predicted
		p73	Ensembl	G1U940 fragment	predicted
Dog	yes	p53	UniProt	Q29537	yes
Canis familiaris		p63	Ensembl	ENSCAFT00000022170	predicted
		p73	Ensembl	ENSCAFT00000030985	predicted
Elephant	yes	p53	UniProt	G3T035	predicted
Loxodonta africana		p63	UniProt	G3TT62	predicted
		p73	UniProt	G3SZA7	predicted
Chicken	yes	p53	UniProt	P10360	yes
Gallus gallus		p63	UniProt	Q9DEC7	yes
		p73	Ensembl	ENSGALT00000001487	predicted[1]
Zebra finch	yes	p53	GenBank	EE060750 (EST)	yes[2]
Taeniopygia guttata		p63	Ensembl	ENSTGUT00000009791	predicted
		p73	Ensembl	ENSTGUT00000002858	predicted
African clawed frog	yes	p53	UniProt	Q6NTF1	yes
Xenopus tropicalis		p63	Ensembl	ENSXETT00000055541	predicted
		p73	Ensembl	ENSXETT00000010981	predicted
Axolotl	no	p53	Uniprot	Q0GMA7	yes
Ambystoma mexicanum		p63	-	-	-
		p73	-	-	-
Coelacanth	yes	p53	UniProt	H3B1Z4	predicted
Latimeria chalumnae		p63	UniProt	H3B2L6	predicted
		p73	UniProt	H3BII1	predicted
Elephant shark	yes	p53	UniProt	G9J1L8	yes
Callorhinchus milli		p63	UniProt	G9J1L9	yes
		p73	UniProt	G9J1M0	yes
Spotted gar	yes	p53	Ensembl	ENSLOCG00000013832[3]	predicted
Lepisosteus oculatus		p63	Ensembl	ENSLOCG00000005148[3]	predicted
		p73	Ensembl	ENSLOCT00000006351	predicted
Zebrafish	yes	p53	UniProt	G1K2L5/Q502Q9	yes
Danio rerio		p63	UniProt	A7YYJ7	yes
		p73	UniProt	Q801Z7	yes
Barbel	no	p53	UniProt	Q9W678	yes
Barbus barbus		p63	-	-	-
		p73	UniProt	Q9W664	yes
Channel catfish	no	p53	Uniprot	O93379	yes
Ictalurus punctatus		p63	-	-	-
		p73	-	-	-
Yellow catfish	no	p53	UniProt	F5A7P3	yes
Tachysurus fulvidraco		p63	-	-	-
		p73	-	-	-
Walking catfish	no	p53	GenBank	GW787457 (EST)	yes
Clarias batrachus		p63	-	-	-
		p73	-	-	-
Atlantic salmon	no	p53	Uniprot	C0H8X1	yes
Salmo salar		p63	-	-	-
		p73	-	-	-

Table S2 (continued)

Accession codes of selected vertebrate p53 family proteins

Species	Genome sequenced	Protein	Database	Accession code	Expression
Rainbow trout	no	p53	Uniprot	P25035	yes
Oncorhynchus mykiss		p63	-	-	-
		p73	-	-	-
European whitefish	no	p53	Uniprot	B5TJK8	yes
Coregonus lavaretus		p63	-	-	-
		p73	-	-	-
Northern pike	no	p53	GenBank	GH247610 (EST)	yes
Esox lucius		p63	-	-	-
		p73	-	-	-
Rainbow smelt	no	p53	GenBank	EL541113 (EST)	yes
Osmerus mordax		-	-	-	-
		-	-	-	-
Atlantic cod	yes	p53	GenBank	GW859200 (EST)[4]	yes
Gadus morhua		p63	Ensembl	ENSGMOT00000000426	predicted[5]
		p73	Ensembl	ENSGMOT00000017093	predicted
Medaka	yes	p53	UniProt	P79820	yes
Oryzias latipes		p63	UniProt	H2MLN6	predicted
		p73	UniProt	H2LHQ7	predicted
Turquoise killifish	no	p53	UniProt	B3TLB0	yes
Nothobranchius furzeri		p63	-	-	-
		p73	-	-	-
Tilapia	yes	p53	UniProt	D5KTJ0 (I3KRX9 error)	yes
Oreochromis niloticus		p63	UniProt	I3KT80	predicted
		p73	UniProt	I3J187	predicted
Southern platyfish	yes	p53	UniProt	Q92143	yes
Xiphophorus maculatus		p63	Ensembl	ENSXMAP00000016461	predicted
		p73	Ensembl	ENSXMAP00000017403	predicted
Three-spined stickleback	yes	p53	UniProt	G3Q6V4	predicted
Gasterosteus aculeatus		p63	UniProt	G3PK82	predicted[4]
		p73	UniProt	G3NTK8	predicted
Mangrove killifish	no	p53	UniProt	A9XR54	predicted
Kryptolebias marmoratus		p63	-	-	-
		p73	-	-	-
Olive flounder	no	p53	UniProt	A5JSV4	yes
Paralichthys olivaceus		p63	-	-	-
		p73	-	-	-
Orange-spotted grouper	no	p53	UniProt	F8RKR1	yes
Epinephelus coioides		p63	-	-	-
		p73	-	-	-
Climbing perch	no	p53	UniProt	R9XXS5	yes
Anabas testudineus		p63	-	-	-
		p73	-	-	-
Pufferfish	yes	p53	UniProt	H2U134	predicted
Takifugu rubripes		p63	UniProt	H2S6K3	predicted
		p73	UniProt	H2UMJ4	predicted
Green spotted puffer	yes	p53	UniProt	H3CXQ0	predicted
Tetraodon nigroviridis		p63	UniProt	H3D8D5	predicted
		p73	UniProt	H3D350	predicted

[1] Prediction of a transcript containing all 14 exons but with a stop codon after residue 213.
[2] Not predicted from current genome assembly
[3] Gene has two transcripts
[4] Missing C-terminal half in the p53 protein predicted from the current version of the genome assembly (ENSEMBL release 74, December 2013)
[5] SAM domain missing

Table S3, related to Figure 2.

Accession codes of p53 family proteins in selected non-vertebrate genomes

Species	Database	Accession code	Expression	Length	SAM domain
Sea squirt *Ciona intestinalis*	UniProt	Q4H300	yes	489	no
	UniProt	Q4H301	yes	415	no
	UniProt	Q4H2Z8[1]	yes	419	no
Lancelet *Branchiostoma floridae*	UniProt	C3YXH3	predicted	416	no
	UniProt	C3XPU2	predicted	649	yes
Soft-shell clam *Mya arenaria*	UniProt	Q9NGC8/Q9NGC7[2]	yes	621/443	yes/no
Blue mussel *Mytilus edulis*	UniProt	Q1AMZ8	yes	657	yes
Pacific oyster *Crassotrea gigas*	UniProt	K1RC48	predicted	790	yes
Sea snail *Lottia gigantea*	Ensembl	LotgiT182533	predicted	607	yes
Bobtail squid *Euprymna scolopes*	UniProt	Q0H3B6	yes	566	yes
Polychaete worm *Capitella teleta*	Ensembl	CapteT137251	predicted	573	yes
Leech *Helbodella robusta*	Ensembl	HelroT167604	predicted	383	no
Sea urchin *Strongylocentrotus purpuratus*	UniProt	H9N2D2/H9N2D3[2]	yes	691/458	yes/no
Roundworm *Caenorhabditis elegans*	UniProt	Q20646	yes	644	yes
Fruit fly *Drosophila melanogaster*	UniProt	Q9N6D8	yes	385	no
Blood fluke *Schistosoma mansoni*	UniProt	G4LYM1	predicted	696	no
	UniProt	G4VAQ1	predicted	410	no
Starlet sea anemone *Nematostella vectensis*	UniProt	A8DPD6	yes	492	no
	UniProt	A7SFL1	predicted	497	no
	UniProt	A7S162	predicted	203	no
Placozoa *Trichoplax adhaerens*	UniProt	B3RZS6	predicted	576	yes
Sponge *Amphimedon queenslandica*	UniProt	I1FGP9	predicted	484	yes
	UniProt	I1FPX8	predicted	332	no
Amoeba *Capsaspora owczarzaki*	UniProt	E9BW10	predicted	730	yes
Choanoflagellate *Monosiga brevicollis*	UniProt	A9V4M3	predicted	571	no
	UniProt	A9UZX3	predicted	523	yes

[1]Gene has no introns

[2]Two different splice variants of a single p63/p73-like gene

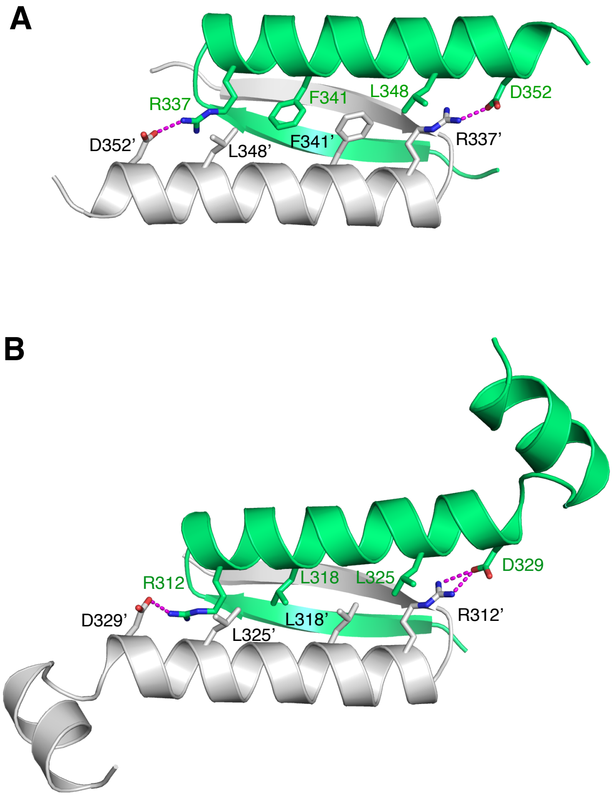

Figure S1, related to Figures 1 and 5. Conserved intermolecular salt bridge in p53 tetramerization domains. (A) Primary dimer in the structure of human p53 tetramerization domain (PDB entry 1C26). The two chains are shown as ribbon diagrams, and selected side chains at the dimer subunit interface are shown as stick models. The second dimer of the tetramer is omitted for clarity. Dimers are stabilized via an intermolecular β-sheet, hydrophobic interactions and a highly conserved salt bridge between Arg337 and Glu352. (B) Primary dimer in the zebrafish p53 tetramerization domain showing conservation of the stabilizing intermolecular salt bridge. The view is the same as in panel A. The central phenylalanine in the human tetramerization domain is replaced by a leucine in the zebrafish protein. Interestingly, there is a slight but systematic deviation from perfect 2-fold symmetry in the packing of the H1 helices within a primary dimer of the zebrafish protein. In all three independent primary dimers of crystal form I, the distance between the Cα atoms of Arg314 and Leu325 from adjacent helices is 7.2 Å at one end of the dimer and 8.7 Å at the other end. Essentially the same difference is also observed in the six independent dimers in the asymmetric unit of crystal form II.

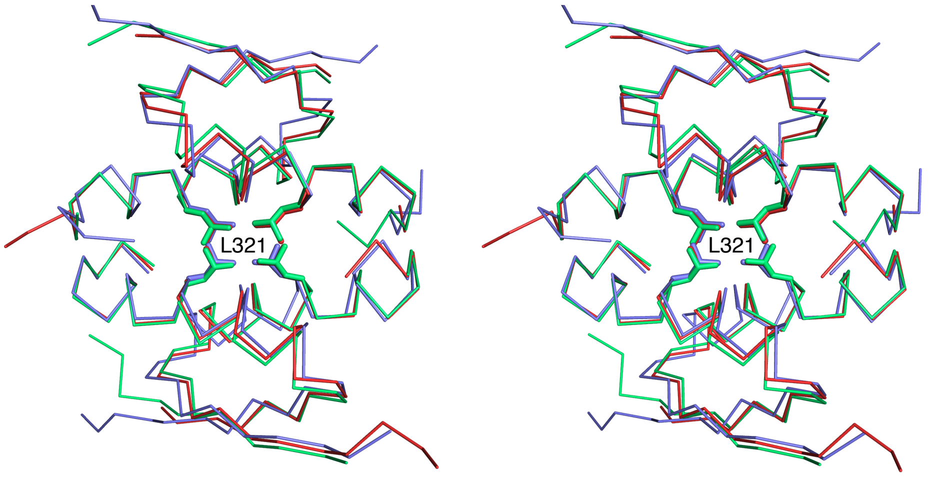

Figure S2, related to Figure 5. Crystal structures of a truncated zebrafish p53 tetramerization domain lacking helix H2. Tetramers observed in three different crystal forms were superimposed and are shown as Cα-traces, with the Leu321 side chains at the center of the tetramer interface shown as stick models. The tetramer in the orthorhombic crystal form I is shown in red, the tetramer in the trigonal crystal form II in green and one of the two tetramers in the monoclinic crystal form III (chains A to D) in blue (see Table 1).
